# Evolution of a σ–(c-di-GMP)–anti-σ switch

**DOI:** 10.1073/pnas.2105447118

**Published:** 2021-07-21

**Authors:** Maria A. Schumacher, Kelley A. Gallagher, Neil A. Holmes, Govind Chandra, Max Henderson, David T. Kysela, Richard G. Brennan, Mark J. Buttner

**Affiliations:** ^a^Department of Biochemistry, Duke University School of Medicine, Durham, NC 27710;; ^b^Department of Molecular Microbiology, John Innes Centre, Norwich NR4 7UH, United Kingdom;; ^c^Département de Microbiologie, Infectiologie et Immunologie, Université de Montréal, Montreal, QC H3T 1J4, Canada

**Keywords:** protein evolution, c-di-GMP signaling, second messenger, *Streptomyces*, RsiG

## Abstract

Filamentous actinobacteria of the genus *Streptomyces* have a complex lifecycle involving the differentiation of reproductive aerial hyphae into spores. We recently showed c-di-GMP controls this transition by arming a unique anti-σ, RsiG, to bind the sporulation-specific σ, WhiG. The *Streptomyces venezuelae* RsiG–(c-di-GMP)_2_–WhiG structure revealed that a monomeric RsiG binds c-di-GMP via two E(X)_3_S(X)_2_R(X)_3_Q(X)_3_D repeat motifs, one on each helix of an antiparallel coiled-coil. Here we show that RsiG homologs are found scattered throughout the Actinobacteria. Strikingly, RsiGs from unicellular bacteria descending from the most basal branch of the Actinobacteria are small proteins containing only one c-di-GMP binding motif, yet still bind their WhiG partners. Our structure of a *Rubrobacter radiotolerans* (RsiG)_2_–(c-di-GMP)_2_–WhiG complex revealed that these single-motif RsiGs are able to form an antiparallel coiled-coil through homodimerization, thereby allowing them to bind c-di-GMP similar to the monomeric twin-motif RsiGs. Further data show that in the unicellular actinobacterium *R. radiotolerans*, the (RsiG)_2_–(c-di-GMP)_2_–WhiG regulatory switch controls type IV pilus expression. Phylogenetic analysis indicates the single-motif RsiGs likely represent the ancestral state and an internal gene-duplication event gave rise to the twin-motif RsiGs inherited elsewhere in the Actinobacteria. Thus, these studies show how the anti-σ RsiG has evolved through an intragenic duplication event from a small protein carrying a single c-di-GMP binding motif, which functions as a homodimer, to a larger protein carrying two c-di-GMP binding motifs, which functions as a monomer. Consistent with this, our structures reveal potential selective advantages of the monomeric twin-motif anti-σ factors.

## Significance

Diverse bacterial lifestyle transitions are controlled by the nucleotide second messenger c-di-GMP, including virulence, motility, and biofilm formation. To control such fundamentally distinct processes, the set of genes under c-di-GMP control must have gone through several shifts during bacterial evolution. Here we show that the same σ–(c-di-GMP)–anti-σ switch has been co-opted during evolution to regulate distinct biological functions in unicellular and filamentous bacteria, controlling type IV pilus production in the genus *Rubrobacter* and the differentiation of reproductive hyphae into spores in *Streptomyces*. Moreover, we show that the anti-σ likely originated as a homodimer and evolved to become a monomer through an intragenic duplication event. This study thus describes the structural and functional evolution of a c-di-GMP regulatory switch.

The nucleotide signaling molecule 3′, 5′-cyclic diguanylic acid (c-di-GMP) is one of the most important nucleotide signaling molecules in bacteria, mediating diverse global processes in response to environmental conditions (1). Cellular levels of this second messenger are determined by the action of diguanylate cyclases (DGCs) that make c-di-GMP from two molecules of GTP, and by c-di-GMP–degrading phosphodiesterases (PDEs) (2–4). DGCs and PDEs are frequently associated with sensory domains, allowing the cell to modulate c-di-GMP levels in direct response to specific stimuli (5). c-di-GMP signaling is best understood in gram-negative bacteria, where it controls processes such as motility, virulence, and biofilm formation (1). Less is known about the roles of c-di-GMP in gram-positive bacteria, but we recently showed that c-di-GMP controls progression through the complex developmental life cycle of the filamentous gram-positive bacteria *Streptomyces* (6–9). *Streptomyces* are ubiquitous soil bacteria that produce numerous secondary metabolites, which serve as our main source of clinically important antibiotics (10). Antibiotic production is temporally and genetically coordinated with morphological development (11, 12), so there is considerable interest in understanding how this progression is determined. The two sets of transcription factors that regulate *Streptomyces* development are encoded by the *bld* (bald) genes, which control the transition from vegetative growth to formation of the reproductive aerial hyphae, and the *whi* (white) genes, which control the differentiation of the reproductive hyphae into chains of exospores (7, 13–15). In *Streptomyces*, c-di-GMP functions as the central integrator of development, directly controlling the activity of two key regulators, BldD and WhiG (6, 9). BldD sits at the top of the regulatory cascade, repressing the transcription of a large regulon of genes, thereby preventing entry into development (6–8, 16). The ability of BldD to repress this set of sporulation genes depends on binding to a tetrameric cage of c-di-GMP that enables BldD to dimerize and thus bind DNA (6–8). As *Streptomyces* enter development, global c-di-GMP levels decrease sharply causing the dissociation of BldD from DNA, and hence the derepression of its regulon.

We recently showed that a second key developmental regulator, the sporulation-specific σ-factor WhiG, is also directly controlled by c-di-GMP. WhiG controls the differentiation of the reproductive aerial hyphae into mature spores (9, 17, 18). Our data revealed that WhiG, which is present throughout the life cycle, is only active at the start of sporulation because it is controlled posttranslationally by a cognate anti-σ, RsiG. Critically, however, RsiG alone cannot sequester WhiG in an inactive complex. Instead, RsiG must bind a partially intercalated dimer of c-di-GMP and only this “nucleotide armed” version of the anti-σ can restrain WhiG (9). Thus, c-di-GMP signals through BldD and WhiG, respectively, to control the two major developmental transitions of the life cycle, the formation of the reproductive aerial hyphae, and their differentiation into spores. In both cases, high levels of c-di-GMP function as a developmental “brake,” blocking further progression through the life cycle (6, 9). Interfering with either of these developmental checkpoints results in dramatic misregulation of the life cycle (6, 9). The structure of the *Streptomyces venezuelae* RsiG–(c-di-GMP)_2_–WhiG complex showed that *S. venezuelae* RsiG is a monomer that binds the partially intercalated dimer of c-di-GMP via two copies of a unique E(X)_3_S(X)_2_R(X)_3_Q(X)_3_D signature motif found on two noncontiguous antiparallel helices, α1 and α5, that form a coiled-coil (9, 19). The conserved residues of the motif, which occur in the same position on the RsiG α1 and α5 helices of its coiled-coil, make symmetric contacts to the two c-di-GMP molecules, and the c-di-GMP dimer itself adopts a symmetrical structure. Binding of c-di-GMP by RsiG promotes the correct conformation of the anti-σ to bind WhiG. The c-di-GMP dimer anchored to the coiled-coil acts as a chaperone, helping to order a long, meandering loop in RsiG, the positioning of which is critical for interaction with WhiG (9). The σ_2_ domain of WhiG itself also makes three contacts with c-di-GMP, and while these are much less extensive than those made by RsiG, they also help to tether the two proteins (9).

RsiG is not homologous to any previously characterized protein and its distribution and evolutionary history in bacteria are therefore unknown. Thus, here we searched for RsiG homologs in representatives from all bacteria and found that its distribution is limited to the phylum Actinobacteria. The c-di-GMP binding motifs among RsiG homologs from diverse actinobacteria are strikingly well conserved, suggesting they likely bind this second messenger. These bacteria also harbor WhiG homologs. Interestingly, however, five of the RsiG homologs that were identified, all from members of class Rubrobacter and class Thermoleophilia, are small proteins that possess a single copy of the c-di-GMP binding motif. Whether these proteins can bind c-di-GMP and function as WhiG anti-σ factors is thus unclear. To address this possibility, we utilized biochemical and structural studies to characterize these single motif-containing RsiG proteins and their complexes with c-di-GMP and WhiG. The structural data, combined with our finding that the sequence similarity between the antiparallel coiled-coil helices of monomeric RsiGs extends beyond the duplication of the E(X)_3_S(X)_2_R(X)_3_Q(X)_3_D c-di-GMP binding motif, suggest that the monomeric anti-σs arose as the result of an internal gene-duplication event, whereby the single-motif containing RsiG proteins can form homodimers to function similar to the twin-motif monomeric RsiGs. Supporting this hypothesis, the five homodimeric single-motif anti-σ factors are found in taxa that are descendants of the most basal branch of the Actinobacterial phylogeny, indicating the single-motif RsiGs represent the ancestral state of this protein. Thus, these studies provide experimental evidence of the evolutionary progression of a symmetric protein. Notably, the actinomycete species that harbor homodimeric single-motif RsiG proteins are unicellular bacteria, raising the question: What does the (RsiG)_2_–(c-di-GMP)_2_–WhiG regulatory switch control in these nonsporulating actinobacteria? Our bioinformatic analysis combined with genome-wide transcription start site mapping suggests that the (RsiG)_2_–(c-di-GMP)_2_–WhiG regulatory switch controls expression of type IV pili in *Rubrobacter*. Overall, these analyses reveal structural and biological details of the evolution of a key c-di-GMP control switch, and how it has evolved to control distinct functions in unicellular and filamentous bacteria.

## Results

### Identification of RsiG Homologs with a Single c-di-GMP Binding Motif.

**Fig. 1.**
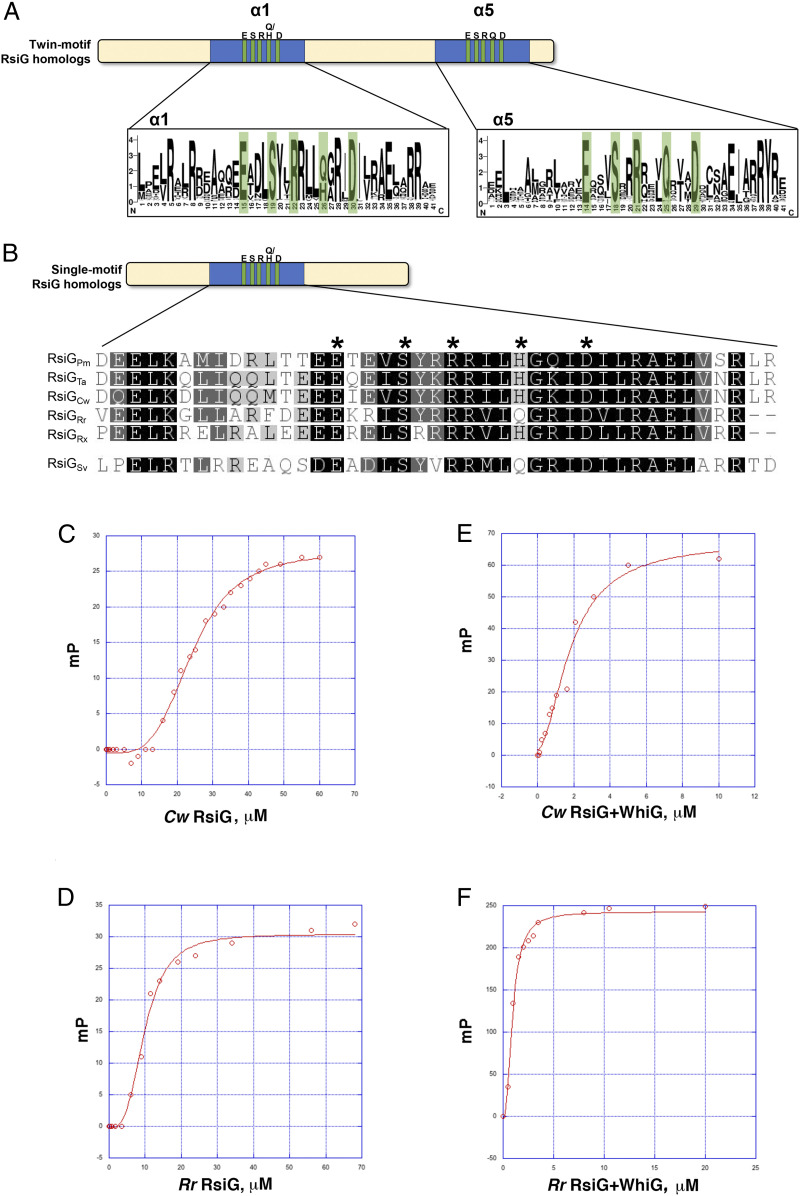
Conservation and binding of c-di-GMP to single-motif RsiG proteins. (*A*) Schematic representation of twin-motif RsiG homologs, such as RsiG from *S. venezuelae* (RsiG_Sv_), above sequence logos depicting amino acid sequence conservation in α1 and α5. Residues that form the c-di-GMP binding motifs are highlighted in green. An alignment including all 130 twin-motif RsiG homologs was used to generate the α1 and α5 logos using WebLogo (50). (*B*) Schematic representation of the single-motif RsiG homologs above an alignment of the sequences of the motif-containing helix from *P. medicamentivorans* (RsiG_Pm_), *T. album* (RsiG_Ta_), *C. woesei* (RsiG_Cw_), *R. radiotolerans* (RsiG_Rr_), and *R. xylanophilus* (RsiG_Rx_). The sequence of the c-di-GMP binding α1 helix from RsiG_Sv_ is shown below for comparison. Residues that form the c-di-GMP binding motif are marked with asterisks. (*C*) Representative binding isotherm of RsiG_Cw_ binding to F-c-di-GMP. (*D*) Representative binding isotherm of RsiG_Rr_ binding to F–c-di-GMP. (*E*) Representative binding isotherm of RsiG_Cw_+WhiG_Cw_ binding to F–c-di-GMP. (*F*) Representative binding isotherm of RsiG_Rr_+WhiG_Rr_ binding to F-c-di-GMP. Three technical repeats were performed for each curve and the SEs from the three affinities were determined. For each panel, the *x* axis and *y* axis show protein concentration in micromolar (μM) and millipolarization units (mP), respectively.

Studies on the interaction of *Streptomyces* RsiG with its cognate σ-factor, WhiG, revealed that RsiG harbors a unique fold for an anti-σ (9). *S. venezuelae* RsiG contains a central antiparallel coiled-coil, in which each helix of the coiled-coil contains a E(X)_3_S(X)_2_R(X)_3_Q(X)_3_D c-di-GMP binding motif. RsiG binds a partially intercalated c-di-GMP dimer with this motif and the bound second messenger mediates interaction with the WhiG σ-protein (9, 19). In addition to the coiled-coil, RsiG contains a linker that connects the coiled-coil helices, an N-tail and a C-terminal region. This RsiG fold and its c-di-GMP binding motif had not been observed previously in any structurally characterized protein. Hence, to identify possible RsiG homologs, we searched a set of 3,962 reference/representative bacterial genomes available at GenBank (673 of which are from the phylum Actinobacteria) using a reciprocal best BLAST search against the *S. venezuelae* RsiG (RsiG_Sv_) sequence as a query. The search revealed 134 RsiG homologs. To identify second copies of RsiG that may exist in these genomes, we further searched within the set of 134 genomes and found that just one, *Acidothermus cellulolyticus* 11B, has a second copy of RsiG, bringing the total number of homologs to 135. RsiG homologs were only found in members of the phylum Actinobacteria, primarily in the families Streptomycetaceae, Geodermatophilaceae, and Pseudonocardiaceae (*SI Appendix*, Table S1). Sequence logos based on a multiple sequence alignment revealed that the two E(X)_3_S(X)_2_R(X)_3_Q(X)_3_D c-di-GMP binding motifs found in RsiG_Sv_ are strikingly well conserved (Fig. 1*A*). The only exception lies in the first motif, found in helix α1, where the glutamine residue present in RsiG_Sv_ is frequently replaced by a histidine in other RsiG homologs. Histidine residues are, however, suitable for making the same stacking interaction with the guanine base of c-di-GMP made by the glutamine residues in the *S. venezuelae* structure (9). Taken together, these results suggest that these RsiG homologs likely bind c-di-GMP.

The notable repetition of the c-di-GMP binding motif raised the possibility that one of the c-di-GMP motif-containing α-helices might have arisen from the other through an intragenic duplication event. In support of this, the sequence identity between the two helices, α1 and α5, is not confined to the E(X)_3_S(X)_2_R(X)_3_[Q/H](X)_3_D motifs but extends across the length of the helices. In RsiG_Sv_, α1 and α5 are 33% identical (*SI Appendix*, Fig. S1) and the identity between these two helices reaches 40% in some other RsiG homologs. Given this finding, it was striking that 5 of the 135 RsiG homologs we identified possess only a single c-di-GMP binding motif. These five sequences were found in the genomes of two members of the class Rubrobacteria, *Rubrobacter radiotolerans* (RsiG_Rr_) and *Rubrobacter xylanophilus* (RsiG_Rx_), and three members of the class Thermoleophilia, *Thermoleophilum album* (RsiG_Ta_), *Patulibacter medicamentivorans* (RsiG_Pm_), and *Conexibacter woesei* (RsiG_Cw_). These proteins harbor the helix equivalent to α1 in RsiG_Sv_ with a conserved c-di-GMP binding motif (Fig. 1*B*), but the helix equivalent to α5 in RsiG_Sv_ is absent in these proteins (*SI Appendix*, Fig. S2).

### Single-Motif RsiG Proteins Form Complexes with WhiG σ-Factors.

Our analyses revealed that bacteria harboring single-motif RsiGs also encode WhiG homologs. To determine if these WhiG proteins interact with the single-motif RsiG proteins from the same species, we co-overexpressed each of the single-motif RsiGs with its cognate WhiG in *Escherichia coli*, with an N-terminal His-tag only on the anti-σ factor. In each case, the single-motif RsiG–WhiG pairs copurified on a nickel column (For example, see *SI Appendix*, Fig. S3). However, extensive washing of the column resulted in unbinding of the WhiG proteins. This was also observed in our previous studies with the twin-motif His-tagged RsiG_Sv_ coexpressed with a non–His-tagged WhiG_Sv_. In those studies, we showed that the RsiG_Sv_–WhiG_Sv_ interaction was dependent on c-di-GMP that copurified with the complex from the *E. coli* expression system. Extensive washing removed the c-di-GMP, resulting in release of WhiG_Sv_ from RsiG_Sv_. Thus, the data suggested that the single-motif RsiG proteins might also bind c-di-GMP to facilitate sequestration of their WhiG partner proteins.

### Single-Motif RsiG Proteins and Their Complexes with WhiG Bind c-di-GMP.

To determine whether the single-motif containing RsiGs can bind c-di-GMP, we next performed fluorescence polarization (FP) studies using the fluoresceinated c-di-GMP probe, 2′-O-(6-[Fluoresceinyl]aminohexylcarbamoyl)-cyclic diguanosine monophosphate (2′-Fluo-AHC–c-di-GMP), which we previously used in binding studies with the RsiG_Sv_ protein (9). For these experiments, we analyzed binding of the probe to RsiG_Rr_, which harbors a glutamine in its putative c-di-GMP binding motif [E(X)_3_S(X)_2_R(X)_3_Q(X)_3_D], and RsiG_Cw_, which has a histidine in this position [E(X)_3_S(X)_2_R(X)_3_H(X)_3_D]. Both proteins bound 2′-Fluo-AHC–c-di-GMP, RsiG_Cw_ with a *K*_d_ of 25 ± 2 µM and RsiG_Rr_ with a *K*_d_ of 12 ± 3 µM (Fig. 1 *C* and *D*). These binding affinities are lower than that obtained for the monomeric, twin-motif RsiG_Sv_, which bound 2′-Fluo-AHC–c-di-GMP with a *K*_d_ of 6.5 ± 1.5 μM, but nevertheless show that these proteins indeed bind c-di-GMP. The binding affinity of RsiG_Sv_ for c-di-GMP was enhanced from 6.5 ± 1.5 μM to 0.39 ± 0.05 µM in the presence of WhiG_Sv_ (9). Therefore, FP analyses of RsiG in the presence of WhiG report on formation of a RsiG–(c-di-GMP)–WhiG complex. Thus, we next measured the binding affinities of RsiG_Rr_+WhiG_Rr_ and RsiG_Cw_+WhiG_Cw_ for 2′-Fluo–AHC-c-di-GMP. Similar to our observations with RsiG_Sv_+WhiG_Sv_, the RsiG_Rr_+WhiG_Rr_ and RsiG_Cw_+WhiG_Cw_ mixtures bound c-di-GMP with ∼15-fold enhanced affinity compared to the RsiG protein alone. Specifically, RsiG_Rr_+WhiG_Rr_ and RsiG_Cw_+WhiG_Cw_ bound 2′-Fluo-AHC–c-di-GMP with *K*_d_s of 0.9 ± 0.05 μM and 1.7 ± 0.3 μM, respectively (Fig. 1 *E* and *F*). Notably, neither RsiG_Cw_ nor RsiG_Rr_, nor their complexes with WhiG bound the identically fluoresceinated c-di-AMP derivative, 2′-O-(6-[Fluoresceinyl]aminohexylcarbamoyl)-cyclic diadenosine monophosphate (2′-Fluo-AHC–c-di-AMP), indicating that the interaction with c-di-GMP is specific.

### Crystal Structures of the *R. radiotolerans* RsiG_Rr_ Protein Reveal a Homodimer.

**Fig. 2.**
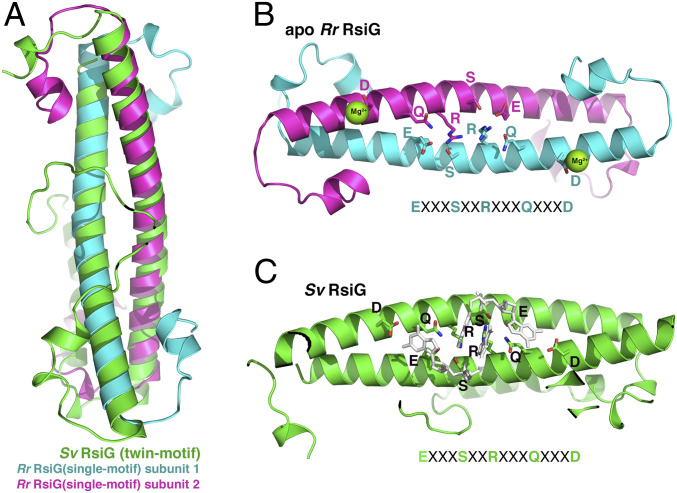
Crystal structure of RsiG_Rr_. (*A*) Overlay of RsiG_Rr_ apo structure homodimeric coiled-coil region (dark magenta and cyan) onto the twin-motif RsiG from *S. venezuelae* (green). Note disparate conformations of the N- and C-terminal regions. (*B*) Ribbon diagram of the apo RsiG_Rr_ (cyan and magenta) with the c-di-GMP binding residues shown as sticks and labeled. Below the structure is the c-di-GMP binding signature motif. (*C*) Shown in the same orientation as *B* is the *Sv* RsiG (green) with its c-di-GMP binding residues shown as sticks and labeled. Also shown as white sticks is the c-di-GMP dimer bound in the *Sv* RsiG–(c-di-GMP)_2_–WhiG complex.

To gain insight into single-motif RsiG protein structure, we carried out crystallization trials. Two well-diffracting crystal forms of the RsiG_Rr_ protein were obtained (*SI Appendix*, *Materials and Methods* and Table S2). Crystal form 1 was solved by selenomethionine single-wavelength anomalous diffraction (SAD) and refined to *R*_work_/*R*_free_ values of 17.3%/20.2% to 1.86-Å resolution (Fig. 2 and *SI Appendix*, Fig. S4*A* and Table S2). The structure contains six molecules of RsiG_Rr_ in the crystallographic asymmetric unit (ASU), which form three essentially identical antiparallel dimers (root mean square deviations of 0.6 Å when comparing Cα atoms of the coiled-coils). Indeed, the structure shows that the single-motif RsiG from *R. radiotolerans* is homodimeric, with helical residues 45 to 83 forming a tight antiparallel homodimeric coiled-coil with overall structural similarity to the monomeric twin-motif RsiG from *S. venezuelae* (Fig. 2). The formation of the homodimeric RsiG_Rr_ coiled-coil buries an extensive 1,904 Å^2^ of protein surface from solvent. This corresponds to a predicted ΔG solvation free-energy gain upon interface formation of −35 kcal/mol, supporting that the homodimer is the physiological relevant form (20). The structure of the second RsiG_Rr_ crystal form was solved by molecular replacement (MR) and refined to *R*_work_/*R*_free_ values of 19.9%/26.8% to 2.55-Å resolution. The same antiparallel homodimer found in crystal form 1 was evident in this structure (*SI Appendix*, Fig. S4*B*).

Strikingly, comparison of the RsiG_Rr_ homodimers with the *S. venezuelae* (*Sv*) monomeric twin-motif RsiG (9) shows that, in addition to harboring overall similarities in antiparallel coiled-coil conformation, RsiG_Rr_ also contains the same arrangement of E(X)_3_S(X)_2_R(X)_3_Q(X)_3_D motifs, one from each helix, as observed in RsiG_Sv_ (Fig. 2 *B* and *C*). The same RsiG_Rr_ crystal forms were obtained in the presence of c-di-GMP under identical crystallization conditions, but no nucleotide was visible in the structures. This appears to be due to the presence of Mg^2+^ ions, which were required for crystallization for both crystal forms. The Mg^2+^ ions in both apo structures are coordinated by D74, located at the C terminus of the E(X)_3_S(X)_2_R(X)_3_Q(X)_3_D motif. In the *Sv* RsiG–(c-di-GMP)_2_–WhiG structure, these aspartic acids bind and specify the splayed-out guanines at the ends of the unusual c-di-GMP dimer bound by RsiG (Fig. 2*C*) when in complex with WhiG. The tightly coordinated Mg^2+^ in the RsiG_Rr_ structure sterically disallows c-di-GMP binding (Fig. 2*B*). The presence of the same homodimers in both RsiG_Rr_ apo crystal structures, though they were obtained under very different conditions, supports that single-motif containing proteins can form the same antiparallel coiled-coil homodimer as observed in the monomeric twin-motif RsiG_Sv_. Importantly, the finding also indicates that c-di-GMP binding is not necessary for dimerization of single-motif RsiG proteins. Indeed, only slight alterations in the coiled-coil helices and side-chain movements in the motifs would position them for c-di-GMP binding (Fig. 2 *B* and *C*). In the *Sv* RsiG–(c-di-GMP)_2_–WhiG structure, RsiG_Sv_ makes extensive interactions to the σ_2_ and σ_4_ domains of WhiG_Sv_ using residues in the long loop that connects the coiled-coil helices and helical regions C terminal to the coiled-coil (9). In contrast, the helices of the coiled-coil in RsiG_Rr_ are from separate but identical subunits. Interestingly, the regions that extend N and C terminal to the homodimeric RsiG_Rr_ central coiled-coils adopt short helical structures, which appear to depend on the crystal packing environment (Fig. 2*A*). These findings suggest that, though flexible, these RsiG residues have strong helical propensity and fold upon binding their partner protein (see next section).

### Overall Structure of the *R. radiotolerans* (RsiG)_2_–(c-di-GMP)_2_–WhiG Complex.

**Fig. 3.**
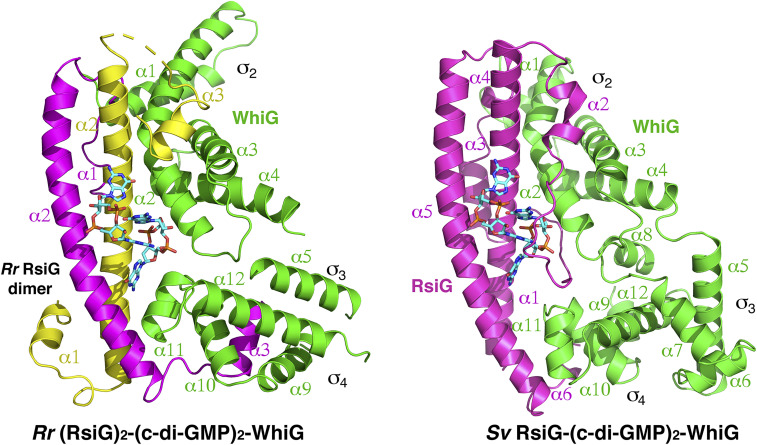
Crystal structure of the *Rr* (RsiG)_2_–(c-di-GMP)_2_–WhiG complex. (*Left*) A ribbon diagram of the *Rr* (RsiG)_2_–(c-di-GMP)_2_–WhiG complex; (*Right*) the *Sv* RsiG–(c-di-GMP)_2_–WhiG complex shown in the same orientation. The WhiG molecules are colored green and their σ_2_ and σ_4_ domains and helices are labeled. The c-di-GMP dimers are shown as sticks. The monomeric twin-motif RsiG_Sv_ is colored magenta. For the homodimeric single-motif RsiG_Rr_ one subunit is colored magenta and the other yellow.

The RsiG_Rr_ structure revealed that this single-motif RsiG protein formed a tight homodimer and our FP data show it binds c-di-GMP. In addition, FP analyses also showed that the combination of the single-motif RsiG and its partner WhiG resulted in significantly enhanced c-di-GMP binding. Collectively, these data support the notion that the single-motif containing RsiG proteins bind c-di-GMP in a similar way to the twin-motif containing RsiG_Sv_. However, it is unclear how RsiG homodimers might interact with their WhiG partners, which are monomeric and contain structurally distinct σ_2_ and σ_4_ domains. Indeed, in the *Sv* RsiG–(c-di-GMP)_2_–WhiG structure the C-terminal region of the RsiG_Sv_ protein, together with the long loop that connects the coiled-coil helices, make distinct contacts to the WhiG_Sv_ σ_2_ or σ_4_ domains. Thus, to elucidate how a single-motif homodimeric RsiG interacts with c-di-GMP and its partner WhiG protein, we solved the crystal structure of the *R. radiotolerans* (*Rr*) RsiG bound to c-di-GMP and Rr WhiG to 2.93-Å resolution. The structure refined to final *R*_work_/*R*_free_ values of 26.5%/27.9% (Fig. 3 and *SI Appendix*, Table S2).

The *R. radiotolerans* complex contains one WhiG_Rr_ molecule, a c-di-GMP dimer, and a RsiG_Rr_ homodimer (Fig. 3). The *Rr* (RsiG)_2_–(c-di-GMP)_2_–WhiG interface is as extensive as the *Sv* RsiG–(c-di-GMP)_2_–WhiG complex, burying 2,545 Å^2^ of protein surface from solvent (compared with 2,353 Å^2^ for the *S. venezuelae* complex). In the complex, the RsiG_Rr_ forms the same antiparallel coiled-coil homodimer as observed in the apo RsiG_Rr_ structures. The domains of WhiG_Rr_ are also structurally similar to the corresponding domains in WhiG_Sv_. WhiG proteins are type 3 σ-factors and thus harbor three domains, with the σ_2_ and σ_4_ domains specifying binding to the promoter −10 and −35 sequences, respectively. RsiG_Rr_ acts as an anti-σ factor by preventing WhiG_Rr_ from binding to RNAP. Comparison of the *Rr* (RsiG)_2_–(c-di-GMP)_2_–WhiG structure with the twin-motif *Sv* RsiG–(c-di-GMP)_2_–WhiG complex, reveals they have the same overall organization, with WhiG_Rr_ positioned comparably against the RsiG_Rr_ coiled-coil (Fig. 3). As in the *Sv* RsiG–(c-di-GMP)_2_–WhiG structure, RsiG_Rr_ interacts with the σ_2_ and σ_4_ regions of WhiG_Rr_ (Fig. 3). However, there are differences between the complexes. For example, the WhiG_Rr_ σ_2_ domain harbors extended α1 and α2 helices. More notably, the WhiG_Rr_ σ_4_ domain is also significantly shifted toward the c-di-GMP dimer in comparison to the *Sv* RsiG–(c-di-GMP)_2_–WhiG structure and, as discussed below, this domain in WhiG_Rr_ makes important contacts to the cyclic nucleotide (Fig. 3). In addition, the *Rr* RsiG homodimeric coiled-coil, though similar to the coiled-coil in the RsiG_Sv_ monomer, lacks the long interfacing loop seen in RsiG_Sv_. Instead, RsiG_Rr_ employs N-terminal and C-terminal extensions from its coiled-coil to bind WhiG_Rr_ (Fig. 3). Consistent with these structural differences between the *R. radiotolerans* and *S. venezuelae* complexes, the *Rr rsiG* gene failed to complement the *Sv rsiG* mutant, even when overexpressed (*Materials and Methods*).

### c-di-GMP Contacts with WhiG_Rr_ and Homodimeric RsiG_Rr_.

**Fig. 4.**
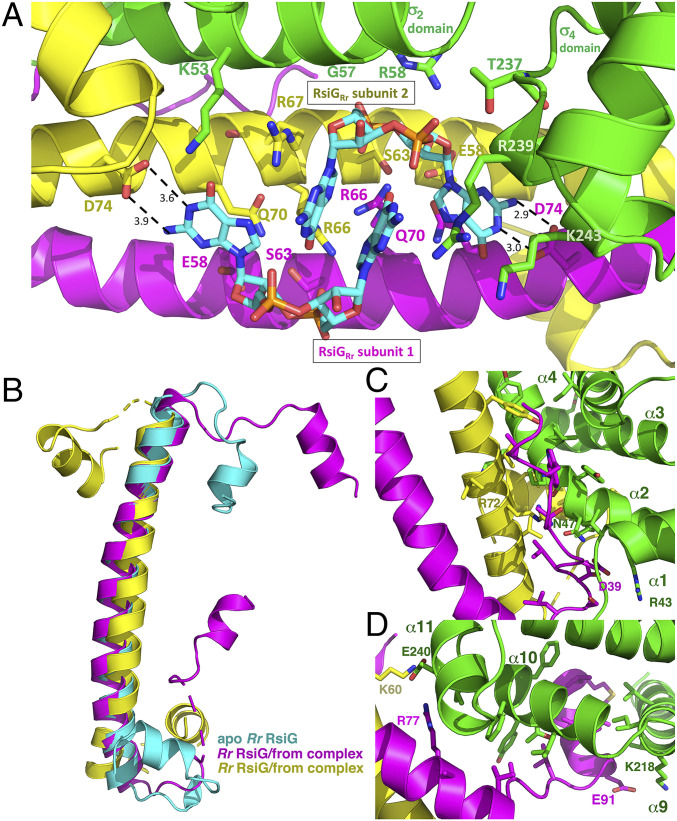
Contacts to c-di-GMP and WhiG_Rr_ from the homodimeric RsiG_Rr_ and flexibility of the WhiG_Rr_ binding regions of RsiG_Rr_. (*A*) Close-up of the c-di-GMP binding region of the RsiG_Rr_ homodimer with one subunit colored yellow and the other magenta. WhiG_Rr_ is green. Residues that contact the c-di-GMP dimer are labeled. (*B*) Overlay of individual RsiG_Rr_ subunits from the WhiG-bound complex (magenta and yellow) and from apo RsiG_Rr_ (cyan), revealing that only the coiled-coil helices are similarly structured. In contrast, the N- and C-terminal extensions adopt distinct conformations upon binding the WhiG_Rr_ σ_2_ and σ_4_ domains. (*C*) Interactions between RsiG_Rr_ and the σ_2_ domain of WhiG_Rr_. (*D*) Interactions between RsiG_Rr_ and the σ_4_ domain of WhiG_Rr_.

Because no c-di-GMP was added prior to crystallization, the c-di-GMP molecules seen in the *Rr* (RsiG)_2_–(c-di-GMP)_2_–WhiG structure copurified with the complex from the *E. coli* expression system (*SI Appendix*, Fig. S5). The bound c-di-GMP dimer is coordinated by two RsiG_Rr_ E(X)_3_S(X)_2_R(X)_3_Q(X)_3_D repeat motifs, with the same arrangement of contacts as observed in the *Sv* RsiG–(c-di-GMP)_2_–WhiG structure (9), but again, in this case each motif is provided by each subunit of the RsiG_Rr_ homodimer (Fig. 4*A*). The conserved serine and glutamic acid residues make hydrogen bonds to the ribose O2′ atom. The conserved glutamines stack with guanine bases. Specificity for guanine nucleotides is provided by the conserved motif residues, aspartic acids, D74, and arginines, R66. The two D74 residues each recognize the Watson Crick faces of the guanines that are splayed out of the c-di-GMP dimer (Fig. 4*A*). Each of the R66 side chains reads one of the stacked guanines located at the center of the c-di-GMP dimer by hydrogen bonding to the guanine O6 and N7 atoms. These contacts provide specificity as well as function to anchor the c-di-GMP dimer in place at the interface between RsiG_Rr_ and WhiG_Rr_.

In the *Sv* RsiG–(c-di-GMP)_2_–WhiG structure, only three contacts were observed between WhiG_Sv_ and the bound c-di-GMP. These interactions are from WhiG_Sv_ residues K57, G61, and R62, located on the α2 helix of the σ_2_ domain. These residues interact with a c-di-GMP guanine O6 atom, a ribose ring, and a phosphate moiety, respectively (9). Our sequence analyses reveal that these c-di-GMP–contacting residues are conserved in WhiG proteins that bind either single-motif or twin-motif RsiG proteins. Thus, as expected, the corresponding contacts from the WhiG_Rr_ σ_2_ domain α2 helix, K53, G57, and R58, are observed in the *Rr* (RsiG)_2_–(c-di-GMP)_2_–WhiG structure (Fig. 4*A*). However, in contrast to the *S. venezuelae* structure, which shows no contacts from the WhiG σ_4_ domain to c-di-GMP, the WhiG_Rr_ σ_4_ domain makes extensive contacts to the c-di-GMP. As noted, in the *R. radiotolerans* structure the WhiG_Rr_ σ_4_ domain is shifted relative to the *S. venezuelae* structure such that it directly abuts the c-di-GMP molecules, positioning residues Thr237, R239, and K243 from the α11 helix of σ_4_ to directly contact the bound c-di-GMP dimer (Figs. 3 and 4*A*). T237 contacts a ribose O4 atom, K243 hydrogen bonds with a guanine O6 atom, while R239 hydrogen bonds with a guanine N7 and stacks with one of the central guanine bases (Fig. 4*A*). In addition, the WhiG_Rr_ σ_4_ α11 N-terminal helix positive dipole is positioned to interact with a c-di-GMP phosphate moiety (Fig. 4*A*).

In its position, α11 also effectively shields the c-di-GMP molecules positioned next to the σ_4_ domain from solvent. As a result, the extended guanine base and region of the c-di-GMP molecules encompassed by WhiG_Rr_ σ_4_ appears more tightly tethered to RsiG than the other end of the c-di-GMP dimer; the hydrogen bond distances from D74 to the N1 and N2 atoms of this guanine are 3.0 Å and 2.9 Å, respectively, compared to 3.6 Å and 3.9 Å from the D74 residue located at the more exposed end of the molecule (Fig. 4*A*). These contacts from WhiG_Rr_ σ_4_ likely partially take the place of the stabilizing interactions made by the long loop present in the twin-motif RsiG_Sv_ protein that shields the entire c-di-GMP dimer in that structure (9). Indeed, overlays of the *S. venezuelae* and *R. radiotolerans* structures show that the σ_4_ domain, which is shifted in the *R. radiotolerans* structure compared to the *S. venezuelae* complex, overlaps with the position of the RsiG_Sv_ loop (*SI Appendix*, Fig. S6). It should be noted, however, that although the loop in the *S. venezuelae* structure comes from RsiG, its folding and formation appear to depend on its interaction with WhiG_Sv_ (9).

### Sequence-Identical Regions of the Homodimeric RsiG_Rr_ Subunits Fold into Different Structures to Bind Different WhiG Domains.

While the binding arrangement of the c-di-GMP dimer and many of the contacts to the cyclic nucleotide are conserved between the *R. radiotolerans* and *S. venezuelae* structures, the *R. radiotolerans* structure reveals that there are more contacts from WhiG_Rr_ to c-di-GMP. These contacts partially compensate for the RsiG loop missing in the single-motif RsiGs. However, the result of the missing loop interactions is that in the single-motif RsiG complex, the c-di-GMP dimer is asymmetrically bound with one end less well anchored (as seen from the weaker hydrogen bonds to the c-di-GMP). Strikingly, this contrasts with the largely symmetrically bound c-di-GMP dimer in the *S. venezuelae* complex structure. In addition, notably different between the two complexes are the interactions between the RsiG and WhiG proteins. The *R. radiotolerans* structure shows this is achieved by the N-terminal and C-terminal regions that extend from the coiled-coils adopting distinct conformations upon WhiG binding; superimposition of the two sequence-identical RsiG_Rr_ subunits, as well as those from the apo RsiG_Rr_ structures, reveals that only the helices of the coiled-coil superimpose, with the extended N- and C-terminal regions displaying very different conformations despite their identical sequences (Fig. 4*B*).

The interface between RsiG_Rr_ and the σ_2_ domain of WhiG_Rr_ involves all helices of the σ_2_ domain, which are encased by the N-terminal extension of one RsiG_Rr_ subunit and the C-terminal extension of the other RsiG_Rr_ subunit (Figs. 3 and 4*C*). Residues from WhiG_Rr_ σ_2_ helix α3 make hydrophobic contacts with the C-terminal helix of one RsiG_Rr_ subunit while WhiG_Rr_ σ_2_ helix α4 interacts with the N-terminal helix of the other RsiG_Rr_ subunit. The C terminus of WhiG_Rr_ α1 inserts within the N-terminal loop that connects RsiG_Rr_ α1 to the coiled-coil helix. The WhiG_Rr_ α2 helix, however, makes most of the contacts with RsiG_Rr_. This extended and bent WhiG_Rr_ helix interfaces not only with the RsiG_Rr_ coiled-coil helix but also interacts with both the N-terminal helix of one RsiG_Rr_ subunit and the C-terminal helix of the other RsiG_Rr_ subunit (Fig. 4*C*). The contacts in this interface are primarily nonspecific and hydrophobic in nature; however, there is one electrostatic interaction in this interface between RsiG_Rr_ residue D39 and WhiG_Rr_ residue R43. In addition, WhiG_Rr_ residue N47 hydrogen bonds to R72 from one RsiG_Rr_ subunit and D39 from the other RsiG_Rr_ subunit (Fig. 4*C*).

The contacts between RsiG_Rr_ and the WhiG_Rr_ σ_4_ region involve only one RsiG_Rr_ subunit. In this interface, the σ_4_ domain helices α9, α10, and α11 interweave with the C-terminal region of an RsiG_Rr_ coiled-coil helix, the loop that follows and the short C-terminal helix α3 (Fig. 4*D*). Again, most of the interactions in this interface are hydrophobic. There are just two sets of electrostatic interactions, one between WhiG_Rr_ K218 and RsiG_Rr_ E91 and the other from WhiG_Rr_ residue E240 to R77 in one RsiG_Rr_ subunit and K60 in the other RsiG_Rr_ subunit.

The *Rr* (RsiG)_2_–(c-di-GMP)_2_–WhiG structure revealed how sequence-identical regions of the two RsiG subunits can flexibly adopt different conformations, allowing them to interact with distinct WhiG_Rr_ σ_2_ and σ_4_ domains. The glycine residues in RsiG_Rr_ that link the coiled-coil helix to the N- and C-terminal extensions appear key in allowing the multiple configurations of these regions for WhiG docking. Indeed, sequence comparisons of single-motif RsiGs revealed they all contain glycine-rich regions that connect their coiled-coil helices to the N- and C-terminal extensions. The RsiG residues that follow the glycine-rich stretch and contact WhiG contain a mixture of hydrophobic and hydrophilic residues. These regions are particularly rich in leucine and glutamic acid residues, which have high helical propensity, thereby facilitating their helical transitions upon WhiG binding. The scattered hydrophobic and hydrophilic residues in these regions enable folding and nonspecific engagement with the σ_2_ or σ_4_ domain of WhiG_Rr_.

### Two-Hybrid Analyses Support that the Single-Motif RsiG Proteins Are Homodimers.

Our structural data showed that the single-motif RsiG_Rr_ protein forms a homodimer in the presence and absence of c-di-GMP, thus explaining how it functions as an anti-σ factor in a c-di-GMP–dependent manner with its partner WhiG protein. To test the structural model in vivo, we assayed all five of the single-motif RsiG homologs (RsiG_Rr_, RsiG_Rx_ RsiG_Ta_, RsiG_Cw_, and RsiG_Pm_) for self-interaction using a bacterial acdenylate cyclase two-hybrid (BACTH) system, exploiting the monomeric twin-motif RsiG from *S. venezuelae* as a negative control. We found that each of the five single-motif RsiG homologs self-interacted, in sharp contrast to the monomeric twin-motif protein, RsiG_Sv_, which did not (*SI Appendix*, Fig. S7). Thus, these data support our structural and biochemical analyses, showing that the single-motif RsiG proteins form homodimers, enabling them to bind c-di-GMP and interact with WhiG σ-factors.

### Distribution of Single- and Twin-Motif RsiG Proteins within the Phylum Actinobacteria.

**Fig. 5.**
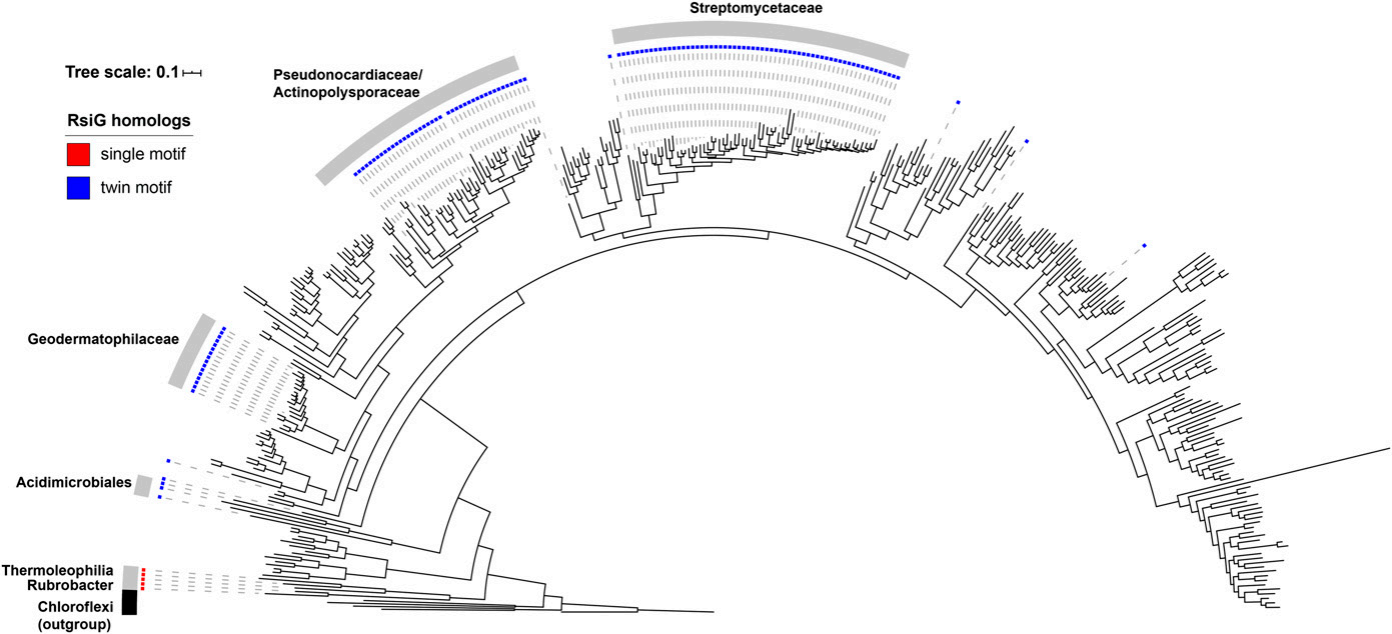
Distribution of RsiG homologs in the phylum Actinobacteria. A maximum-likelihood phylogeny of 378 representative Actinobacterial species is shown, based on 37 concatenated housekeeping genes that were identified and aligned using PhyloSift (51). Sequences derived from five Chloroflexi genomes were used to root the tree (indicated in black). Genomes possessing an RsiG homolog are indicated by colored boxes, with red boxes signifying the presence of homodimeric RsiG homologs (with a single c-di-GMP binding motif), and blue boxes signifying the presence of monomeric RsiG homologs (with two c-di-GMP binding motifs). Major taxonomic groups with at least two representatives in which an RsiG homolog is found in >80% of genomes are indicated by the gray arcs. Tree scale is substitutions per site. A version of this tree that includes full taxonomic labeling and node support values can be found in *SI Appendix*, Fig. S8.

To examine the phylogenetic distribution of the two evolutionary states of RsiG that we have identified, monomeric single-motif RsiGs and dimeric twin-motif RsiGs, we constructed a housekeeping phylogeny that reflects the evolutionary history of the 673 Actinobacterial genomes that were included in the initial search for RsiG homologs. For simplicity, genera in the tree that claded monophyletically and had no RsiG homologs were further pruned, retaining at least two representatives per genus, bringing the total number of Actinobacterial taxa in the tree to 378 (Fig. 5 and *SI Appendix*, Fig. S8). The phylogeny reveals that class Rubrobacter and class Thermoleophilia, which are the only clades that possess dimeric single-motif RsiG homologs, are sister taxa that evolved from an early branch of the phylum Actinobacteria. This distribution is consistent with the hypothesis that the dimeric single-motif form of RsiG is ancestral, and the monomeric twin-motif form of RsiG is the evolutionarily derived state. The twin-motif form of RsiG is most highly represented in members of the Streptomycetaceae (62 of 62 species), where it was first discovered, the Geodermatophilaceae (16 of 16 species), the Pseudonocardiaceae (39 of 46 species), the Actinopolysporaceae (2 of 2 species), and the Acidimicrobiales (3 of 4 species). This suggests that a combination of vertical inheritance and gene loss resulted in the distribution of the monomeric twin-motif RsiG homologs that we observe today (Fig. 5 and *SI Appendix*, Fig. S8). In addition, isolated representatives of the twin-motif form are also found scattered elsewhere in the Actinobacterial phylogeny, likely representing horizontal gene-transfer events (Fig. 5 and *SI Appendix*, Fig. S8).

### The (RsiG)_2_–(c-di-GMP)_2_–WhiG Regulatory Switch Controls the Production of Type IV Pili in the Genus *Rubrobacter*.

Our structural and biochemical analyses show how the single-motif RsiG proteins are able to bind c-di-GMP and function as anti-σ factors, and combined with our phylogenetic analyses, suggest how these proteins may have evolved to give rise to the twin-motif proteins inherited elsewhere in the Actinobacteria, including the genus *Streptomyces* where RsiG was first characterized (9). An important additional question concerns the evolution of the biological function of the RsiG–(c-di-GMP)–WhiG switch. In *Streptomyces*, this switch is a dedicated component of the regulatory cascade controlling the differentiation of the reproductive hyphae into spores. However, the five sequenced actinomycete species that contain single-motif RsiG proteins are nonsporulating, unicellular bacteria (21–26).

**Fig. 6.**
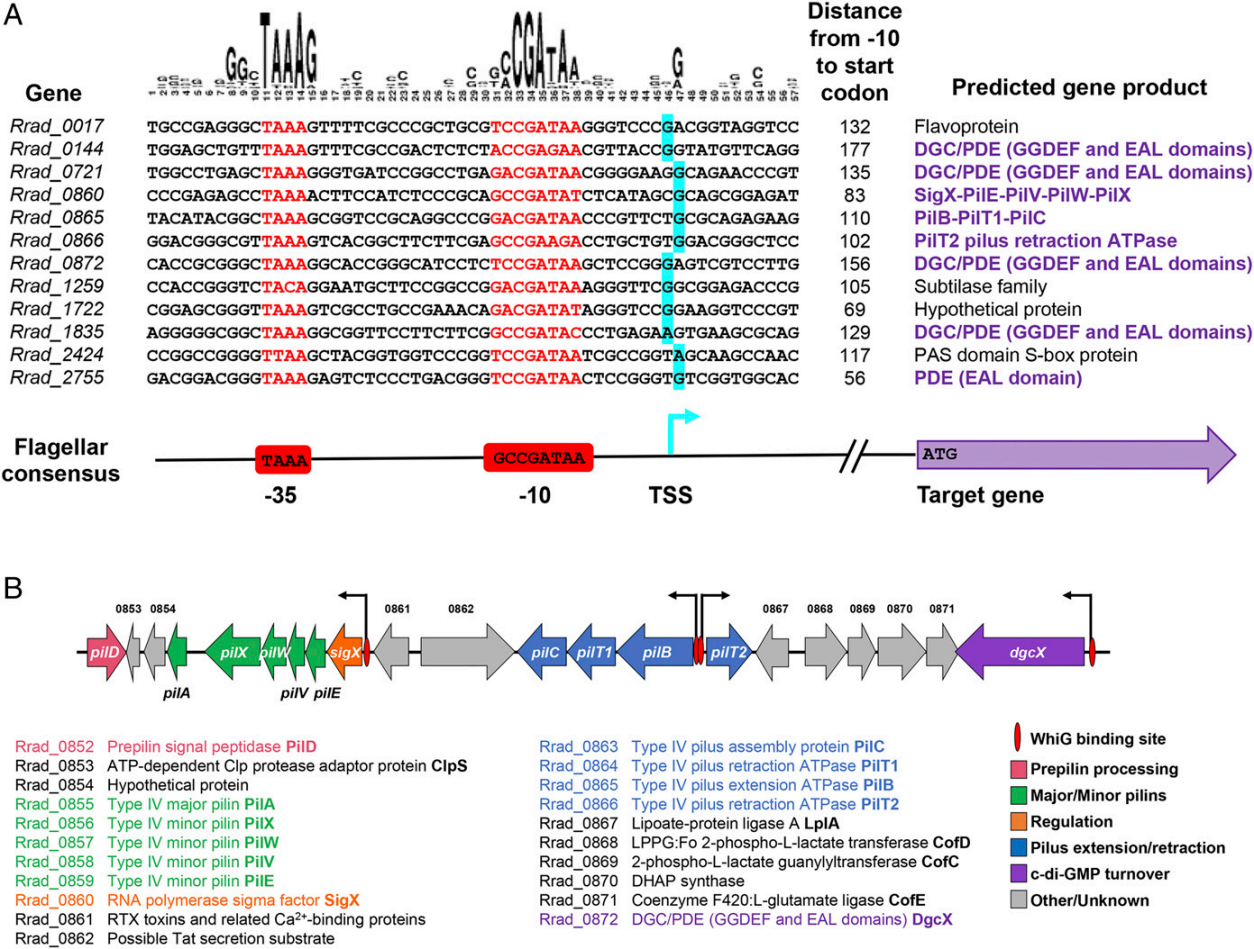
Predicted WhiG target promoters in *R. radiotolerans* and organization of the *R. radiotolerans* type IV pilus gene cluster, showing the positions of predicted WhiG target promoters. (*A*) Twenty matches to the well-established flagellar promoter consensus sequence (−35 TAAA; −10 GCCGATAA) (27) were identified bioinformatically in the intergenic regions of the *R. radiotolerans* genome, lying within 200 bp of a downstream start codon, and allowing for up to two base mismatches in total. Of these 20 sequences, the 12 shown were found to sit just upstream of appropriately positioned transcription start sites, demonstrating that they represent genuine promoters. Transcription start sites (TSS), shown in blue, were determined as part of a genome-wide 5′ triphosphate end-capture transcription start site mapping experiment. Putative −10 and −35 sequences are shown in red. Target genes with predicted functions in type IV pilus biosynthesis or c-di-GMP turnover are highlighted in purple. The logo based on the sequence alignment was created using Weblogo (50). (*B*) The genes in the type IV pilus gene cluster are shown as schematics with the predicted gene products listed below with the same color coding.

To gain insight into the biological function of the structurally characterized *Rr* (RsiG)_2_–(c-di-GMP)_2_–WhiG switch, we carried out bioinformatic searches for likely WhiG target promoters, to see which genes WhiG might control in *R. radiotolerans*. Although WhiG is a dedicated sporulation σ in *Streptomyces*, phylogenetically it is a member of the flagellar clade of σ-factors, with which it shares the same promoter specificity (9). We therefore searched for matches to the well-established “flagellar” promoter consensus sequence (−35 TAAA; −10 GCCGATAA) (27) in the intergenic regions of the *R. radiotolerans* genome, lying within 200 bp of a downstream start codon, and allowing for up to two base mismatches in total. This analysis identified 20 matches. To determine which of these sequences represented in vivo promoters, we isolated RNA from *R. radiotolerans* and subjected it to genome-wide 5′ triphosphate end-capture transcription start site mapping. Twelve of the bioinformatically predicted WhiG target sequences sat just upstream of appropriately positioned transcription start sites, showing that they represent genuine promoters (Fig. 6*A*).

Three of these 12 promoters lie within the type IV pilus gene cluster of *R. radiotolerans*, likely directing expression of nine *pil* genes in three transcription units (Fig. 6*B*). Five of the other promoters identified sit in front of genes encoding DGC/PDE enzymes, one of which (*Rrad_0872*) is closely linked to the type IV pilus gene cluster (Fig. 6). In support of this analysis, when we repeated the search for putative WhiG target promoters in the intergenic regions of the *R. xylanophilus* genome, we obtained similar results. The same bioinformatic search (−35 TAAA; −10 GCCGATAA; up to two base mismatches in total) identified 11 matches (*SI Appendix*, Fig. S9*A*). Although *R. xylanophilus* and *R. radiotolerans* are divergent species, sharing just 74.5% average nucleotide identity (https://img.jgi.doe.gov/mer/), two of the 11 putative promoters in *R. xylanophilus* were again found in the type IV pilus gene cluster, this time potentially directing expression of 13 *pil* genes in two operons (*SI Appendix*, Fig. S9*B*). A third putative WhiG target promoter in *R. xylanophilus* sits in front of a gene encoding a DGC/PDE enzyme (*SI Appendix*, Fig. S9*A*). In addition, *dgcX*, a gene encoding a DGC/PDE enzyme, is embedded within the type IV pilus gene cluster (*SI Appendix*, Fig. S9*B*).

## Discussion

The RsiG–WhiG cognate pair is the only known example of a σ–anti-σ complex that is targeted by c-di-GMP (9, 19). The presence of RsiG homologs throughout the Actinobacteria, including descendants of some of the most basal branches of the phylum, as well as the high degree of conservation of the c-di-GMP binding motif among homologs, indicates that this RsiG–(c-di-GMP)–WhiG switch is ancient and must have appeared during the early evolution of the Actinobacteria some 2 billion y ago (28, 29). The work presented here sheds light not only on the evolution of RsiG as a c-di-GMP binding anti-σ factor, but also on how the RsiG–(c-di-GMP)–WhiG regulatory switch seems to have been co-opted during evolutionary history to control distinct biological functions in unicellular and filamentous bacteria.

In RsiG_Sv_, two copies of the E(X)_3_S(X)_2_R(X)_3_Q(X)_3_D signature repeats are provided by the two helices of its central antiparallel coiled-coil. Similarity between the helices is not limited to the residues in the motifs themselves, which raised the possibility that they are the result of an intragenic gene-duplication event. It was therefore very striking to find that the present-day descendants of the most basal branch of the Actinobacteria, namely class Thermoleophilia and class Rubrobacteria, harbor small RsiG homologs with only a single c-di-GMP binding motif. Consistent with these single-motif RsiG homologs acting as anti-σ factors, we showed that WhiG homologs are also present in these organisms and can indeed bind the single-motif RsiG proteins. Structural analyses revealed that these single-motif RsiGs form an antiparallel coiled-coil through homodimerization, enabling them to bind c-di-GMP in the same manner as the monomeric twin-motif RsiGs. This also allows them to function as anti-σ factors with adjustments in the way they bind their partner WhiG σ-factors.

In nonmotile, filamentous *Streptomyces*, the RsiG–(c-di-GMP)_2_–WhiG regulatory switch controls the differentiation of the reproductive aerial hyphae into spores (9). The present study suggests that this σ–anti-σ switch controls a completely distinct biological process in a nonsporulating, unicellular actinomyete. Specifically, bioinformatic analysis combined with 5′ triphosphate end-capture transcription start site mapping suggest that the structurally characterized (RsiG)_2_–(c-di-GMP)_2_–WhiG switch controls the expression of type IV pili in *R. radiotolerans*. Type IV pili can be involved in diverse functions in bacteria, including motility on solid surfaces, biofilm formation, adhesion to host cells, DNA uptake, and electron transfer (30, 31). In *Myxococcus*, c-di-GMP is important for type IV pili-dependent motility, with high c-di-GMP inhibiting transcription of the major pilin gene, *pilA*, possibly by binding directly to the NtrC-like transcriptional regulator, PilR (32). In *Vibrio cholerae*, biofilm formation is regulated by c-di-GMP and requires production of type IV pili (33, 34). Pilus extension is directly controlled by c-di-GMP, which acts as an effector ligand to regulate the activity of MshE, the extension ATPase that promotes pilus polymerization (35). Within the actinomycetes, type IV pili are known to be present on the surface of the highly unusual motile zoospores of the sporulating, filamentous actinomycete *Actinoplanes missouriensis*, where they are required for efficient adhesion of the zoospores to hydrophobic solid surfaces (36). *Actinoplanes* zoospores possess not only type IV pili but also flagella, and transcription of both the type IV pili genes and the flagella genes is directed by WhiG-like flagellar σ-factors (36–38). However, although *Actinoplanes* species have WhiG, they lack RsiG (Fig. 5 and *SI Appendix*, Fig. S8). The same analyses also suggest that the *Rr* (RsiG)_2_–(c-di-GMP)_2_–WhiG switch directs transcription of multiple *dgc/pde* genes. If this is true, then in *Rubrobacter* this switch not only senses cellular c-di-GMP levels, it may also control the expression of enzymes that synthesize and degrade c-di-GMP, creating potential feedback loops. This would be reminiscent of BldD, the master repressor of sporulation in *Streptomyces*: the ability of BldD to bind DNA is controlled by complex formation with c-di-GMP, and four of the direct targets of BldD-mediated repression are the genes that encode the DGCs CdgA, CdgB, CdgC, and CdgE (6, 8, 16, 39–41).

Many proteins, such as RsiG, exhibit elements of internal structural symmetry. Gene duplication and fusion of peptide ancestors is one mechanism, along with convergent evolution, hypothesized to be responsible for their emergence (42, 43). To date, most studies performed to assess evolutionary models of protein folds have been carried out on small peptide motifs and the results generally support the gene-duplication and fusion mechanism (44). Within this theory there is, however, debate as to how the symmetric folds have arisen and two distinct models have been proposed: “conserved architecture” and “emergent architecture.” The conserved architecture model posits that small peptide ancestors of symmetric proteins formed oligomers that resemble the eventual fusion protein, while in the emergent architecture model the small peptides do not oligomerize (42, 43). A study utilizing small peptides that can be linked to form a β-trefoil structure was most consistent with the conserved architecture model (44). However, studies are lacking that involve experimental data following the evolutionary progression of a protein fold. Collectively, our biochemical, cellular, and structural studies provide support that the ancestral RsiG bears a single c-di-GMP binding motif, and an intragenic duplication event led to the appearance of twin-motif homologs of RsiG. Moreover, they support a model in which the ancestral RsiG formed homodimers resembling the monomeric twin-motif RsiGs inherited elsewhere in the Actinobacteria.

Twin-motif RsiGs have several potential advantages over single-motif RsiGs that may explain selection for this form of the protein. In particular, the twin-motif proteins do not have to locate and bind a second subunit to form a functional homodimer. In addition, the function of RsiG as an anti-σ factor requires it to bind a highly asymmetric monomeric σ-protein. Thus, even though the regions connected to the RsiG coiled-coils appear to be flexible in both the single- and twin-motif proteins, the single-motif containing RsiG homologs must utilize these regions to interact with what are, in essence, different binding partners, the σ_2_ and σ_4_ domains of WhiG. In contrast, the twin-motif RsiG homologs use dedicated regions outside the coiled-coil to interact with the WhiG σ_2_ and σ_4_ domains. A loop also appeared during the evolution of the twin-motif RsiGs, which effectively shields the c-di-GMP dimer. The absence of this loop is only partially compensated for in the single-motif RsiG containing complex by contacts to the c-di-GMP from the σ_4_ region of its WhiG partner. Thus, these studies provide a detailed analysis of the evolutionary progression of a symmetric protein, which functions as a novel biological switch, via an internal gene duplication event. Furthermore, these studies also demonstrate that this switch has also evolved to control strikingly different biological functions in unicellular and filamentous bacteria.

## Materials and Methods

### Bacterial Strains, Plasmids, and Media.

Strains, plasmids, and oligonucleotides used in this study are listed in *SI Appendix*, Table S3. *E. coli* strain DH5α was used for plasmid propagation and grown on LB or LB agar at 37 °C. Where required for selection, the following antibiotics were added to growth media: 100 μg/mL carbenicillin, 25 μg/mL chloramphenicol, and/or 50 μg/mL kanamycin.

### Bacterial Two-Hybrid Analysis to Assay RsiG Homodimerization.

*E. coli* codon-optimized versions of the rsiG genes from *C. woesei* (*Cw*), *P. medicamentivorans* (*Pm*), *R. radiotolerans *(*Rr*), *R. xylanophilus* (*Rx*), and *T. album* (*Ta*) were synthesized and cloned into pUC57 (GenScript). These were PCR-amplified with BACTH forward and reverse primers and cloned into pUT18 and pKT25 (45) using the restriction enzymes XbaI and KpnI. *E. coli* BTH101 was then cotransformed with the “T18” and “T25” fusion plasmids. pUT18 and pKT25 constructs expressing the monomeric twin-motif RsiG from *S. venezuelae* were used as a negative control. β-Galactosidase activity was assayed in triplicate (biological replicates).

### WhiG-RsiG Cooverexpression Constructs.

Each of the *E. coli* codon-optimized *rsiG* genes described above was amplified using MCS1 forward and reverse primers and cloned into the MCS1 of pCOLADuet1 using the restriction enzymes EcoRI and HindIII. *E. coli* codon-optimized versions of the corresponding *whiG* genes (GenScript) were then amplified with MCS2 forward and reverse primers and cloned into the MCS2 of the pCOLADuet1 derivative carrying the cognate *rsiG* gene, using the restriction enzymes NdeI and KpnI.

### Small-Scale Purification of RsiG–WhiG Complexes.

*E. coli* carrying pCOLADuet-1 RsiG–WhiG coexpression constructs was grown at 37 °C in 50 mL LB medium with antibiotics to an OD_600_ of 0.45, then induced with 1 mM isopropyl β-d-thiogalactopyranoside (IPTG) at 37 °C overnight. Cells were harvested by centrifugation, resuspended in 1 mL Equil Buffer (Sigma-Aldrich) and lysed by sonication. Cell debris was removed by centrifugation and RsiG–WhiG complexes were purified on HIS-Select Spin columns (Sigma-Aldrich).

### Large-Scale Purification of RsiG_Cw_, RsiG_Rr_, RsiG_Cw_–WhiG_Cw_, and RsiG_Rr_–WhiG_Rr_.

C41(DE3) cells were transformed with RsiG_Cw_ (cloned into pET15b), RsiG_Rr_ (cloned into pET15b), RsiG_Cw_–WhiG_Cw_ (cloned into pCOLADuet-1), and RsiG_Rr_–WhiG_Rr_ (cloned into pCOLADuet-1) expression vectors (*SI Appendix*, Table S3). The full-length (FL) RsiG_Cw_ protein was expressed in apo form and in the presence of WhiG_Cw_ for biochemical and structural studies. The FL RsiG_Rr_ is comprised of 118 residues. Two constructs were expressed and purified for structural studies, FL RsiG_Rr_, and RsiG_Rr_(27-106). The latter construct was generated based on the findings of an initial FL RsiG_Rr_ structure showing that N- and C-terminal residues were disordered (see below). Genes expressing FL RisG_Cw_, FL RsiG_Rr_, and RsiG_Rr_(27-106) were subcloned into pET-15b, between the NdeI and BamHI sites for expression. The resulting proteins harbor cleavable hexa-histidine tags. For protein expression, cells with each expression construct were grown at 37 °C in LB medium with 0.17 μg/mL ampicillin to an OD_600_ of 0.6, then induced with 0.50 mM IPTG at 15 °C overnight. Cells were harvested by centrifugation, then resuspended in Buffer A [50 mM Tris⋅Cl pH 7.5, 300 mM NaCl, 5% glycerol, 0.5 mM β-mercaptoethanol (βME)], with added 1× protease inhibitor mixture and DNase I (1 μg/mL) and disrupted with a microfluidizer. Cell debris was removed by centrifugation (15,000 rpm, 4 °C, 45 min). The supernatant was loaded onto a cobalt NTA column. The column was washed with 300 mL of buffer A and eluted in steps with 5, 20, 30, 40, 50 , 100, 200 mM imidazole in buffer A. Fractions were analyzed by SDS/PAGE and those containing the protein were combined and subjected to thrombin digestion overnight at 37 °C using a thrombin cleavage capture kit (Novagen). The cleaved products were loaded onto a Ni-NTA column and the flow through (in buffer A), which contained the His-tag free protein, collected. The proteins were >95% pure after this step and were concentrated using centricons with a 10-kDa molecular mass cutoff (Millipore).

For RsiG_Cw_–WhiG_Cw_ and RsiG_Rr_–WhiG_Rr_ purification, cells with the expression construct were grown at 37 °C in LB medium with 50 μg/mL kanamycin and 50 μg/mL chloramphenicol to an OD_600_ of 0.45 and induced with 0.50 mM IPTG at 15 °C overnight. Cells were harvested by centrifugation, resuspended in buffer A with added 1× protease inhibitor mixture and DNase I (1 μg/mL) and disrupted with a microfluidizer. Cell debris was removed by centrifugation (15,000 rpm, 4 °C, 45 min). The supernatant was loaded onto a cobalt NTA column. The column was washed with a minimal volume of 40 mL buffer A, after which the complex was eluted in steps with 10, 20, 30, 50, 100, 200, 500 mM imidazole in buffer A. Fractions were analyzed by SDS/PAGE and those containing the complex were combined and the His-tag on the RsiG protein was cleaved using a thrombin cleavage capture kit. The complexes were >90% pure after this step and were concentrated using centricons with 50-kDa cutoff, which also removed the His-tags.

### Crystallization of RsiG_Rr_ and the RsiG_Rr_–(c-di-GMP)–WhiG_Rr_ Complex.

For crystallization of the FL RsiG_Rr_, the protein was concentrated to 25 mg/mL Wizard I to IV, Peg/Ion, and Hampton Screen 1 were used for crystallization screening via the hanging-drop vapor diffusion method at room temperature. Crystals of FL RsiG_Rr_ were obtained by mixing the protein 1:1 with a solution of 0.1 M 4-(2-hydroxyethyl)-1-piperazineethanesulfonic acid (Hepes) pH 7.5, 25% PEG 400, 0.2 M MgCl_2_. Crystals took from 1 to 2 wk to grow to optimal size. The crystals could be cryopreserved straight from the drop. Data were collected on beamline 8.3.1 at the Advanced Light Source (ALS) Berkeley, CA to 3.3-Å resolution. An initial MR solution using the coiled-coil domain of the *Sv* RsiG protein was obtained using the data. After refinement and fitting the structure, density was missing for residues 1 to 26 and the last 10 residues. Hence, to obtain better crystals, the RsiG_Rr_(27-106) construct was generated. For crystallization, purified RsiG_Rr_(27-106) protein was concentrated to 25 mg/mL and the same sets of screens were employed as for the FL protein. Crystals were obtained at room temperature by mixing the protein (±10 mM c-di-GMP) at a ratio of 1:1 with crystallization reagents consisting of either 0.1 M Imidazole pH 8.0, 12% isopropyl alcohol and 0.1 M MgCl_2_ (crystal form 1) or 0.1 sodium acetate pH 4.5, 2.5 M NaCl, 0.2 M lithium sulfate and 0.1 M MgCl_2_ (crystal form 2). Both crystal forms were cryopreserved by dipping them for 1 to 2 s in the crystallization solution supplemented with 25% glycerol. For structural studies the RsiG_Rr_–WhiG_Rr_ was concentrated to 25 mg/mL and subjected to crystallization via hanging-drop vapor diffusion at room temperature using the same screens as described above. Crystals grew after a week in a condition in which the complex was mixed 1:1 with a crystallization solution consisting of 0.1 M Tris pH 8.0, 0.2 M MgCl_2_, 23% PEG 3350. To cryoprotect the crystals before direct placement in the cryostream at the ALS beamline 8.3.1, the crystals were dipped for 1 to 2 s in the crystallization solution supplemented with 20% glycerol.

### RsiG_Rr_ and the RsiG_Rr_–(c-di-GMP)–WhiG_Rr_ Complex Structure Determination.

MR using the low-resolution FL RsiG_Rr_ structure produced solutions for the RsiG_Rr_(27-106) structure. However, to obtain optimal phases for RsiG_Rr_(27-106) structure determination, selenomethionine (semet) SAD was employed. RsiG_Rr_(27-106) contains just two methionines, with one at the N terminus of the protein. Thus, to enhance the selenomethionine signal, RsiG_Rr_(27-106) (L52M-I69M) was constructed. The RsiG_Rr_(27-106) (L52M-I69M) protein was purified as per the WT protein and successfully produced the same crystal forms 1 and 2 as the WT protein. SAD data for crystal form 1 of a selenomethionine substituted RsiG_Rr_(27-106) (L52M-I69M) crystal was collected at beamline 5.0.2 and the data processed with XDS. Autosol in Phenix was used to locate selenium sites, perform heavy atom refinement, and carry out density modification (46). Using the semet sites as a guide, the model was readily traced into the electron density map. The final model includes residues 28 to 98 of the six subunits in the ASU, which combine to form 3 nearly identical dimers (in 3 subunits, residues left over from His-tag cleavage at the N terminal were also visible), 457 water molecules, and 6 Mg^2+^ ions. MolProbity analyses placed the structure in the top 98% of structures solved to a similar resolution. One of the RsiG_Rr_(27-106) dimers was used in MR to obtain an initial solution for crystal form 2. The model produced one clear solution and was used as a static model to find a single RsiG_Rr_(27-106) subunit, which generates a dimer with itself via crystallographic symmetry. The final model includes residues 28 to 98 for the 3 RsiG_Rr_(27-106) subunits, 2 sulfate molecules, 3 Mg^2+^ ions, and 10 water molecules. MolProbity analyses placed it in the top 98% of structures solved to a similar resolution (47). See *SI Appendix*, Table S2 for relevant data collection and refinement statistics for both structures. Data were collected on the same crystal forms of RsiG_Rr_(27-106) produced in the presence of c-di-GMP. However, the nucleotide was not present in the structures.

The RsiG_Rr_–(c-di-GMP)–WhiG_Rr_ crystals take the orthorhombic space group, P2_1_2_1_2_1_ and diffract to 2.93 resolution. Data were collected at beamline 8.3.1 and the data processed with XDS (48). The RsiG_Sv_ coiled-coil successfully produced an MR solution with MolRep. Using this structure as a static starting model permitted the WhiG_Sv_ σ_2_ domain to be placed. Subsequently, the σ_4_ domain of WhiG_Sv_ was successfully fit. The starting model was subjected to a few rounds of *xyz* refinement in Phenix (46) after which the *R. radiotolerans* residues were substituted and the structure refined. After Phenix refinement with the correct side chains the *R*_free_ dropped from 46 to 35%. Density for only part of the σ_3_ domain was observed and its register was unclear. Thus, the residues in this region were modeled as polyalanine. The final model includes WhiG_Rr_ residues 2 to 115' 202 to 271, 2 c-di-GMP molecules, and residues 23 to 99 of one RsiG_Rr_ subunit and residues 28 to 101 of the other subunit.

### Determination of the Affinity and Specificity of c-di-GMP for RsiG and RsiG–WhiG Homologs by FP.

To measure c-di-GMP binding to single-motif RsiG and single-motif RsiG proteins complexed with their WhiG partner proteins, 2′-Fluo-AHC–c-di-GMP (BioLog), was used as a fluoresceinated reporter ligand. This molecule is conjugated by a nine-atom spacer to one of the 2′ hydroxyl groups of c-di-GMP and was chosen as the structure shows that one ribose hydroxyl from each c-di-GMP is solvent exposed when bound to RsiG and the RsiG–WhiG complex and thus not impede binding. The 2′-Fluo-AHC–c-di-AMP) (BioLog) was also used in binding studies to determine the specificity of RsiG and RsiG–WhiG complexes for c-di-GMP. The experiments were all carried out in a buffer of 25 mM Tris⋅HCl pH 7.5 and 150 mM NaCl, which contained 1 nM 2′-Fluo-AHC–c-di-GMP or 2′-Fluo-AHC–c-di-AMP at 25 °C. Increasing concentrations of RsiG or RsiG–WhiG mixtures were titrated into the reaction mixture to obtain their respective binding isotherms. The resultant data were plotted using KaleidaGraph and the curves fit to deduce binding affinities. Note, each batch of these *E. coli*-produced proteins had some c-di-GMP contaminant, which efforts were made to remove, however, variability between batches was noted. Four to three technical repeats were performed for each curve and the SEs from the three affinities were determined.

### RsiG Homolog Identification and Alignment.

RsiG homologs were identified by a reciprocal BLAST search (e-value cutoff = 0.001) of the 3,962 “reference” or “representative” annotated genomes available at GenBank, using the RsiG_Sv_ sequence as a query. Homologs were aligned using MUSCLE (49). Fourteen sequences (all <41% sequence identity) that did not align well were removed from the analysis. To identify genomes with multiple RsiG homologs, a second BLAST search was performed for each of the 134 genomes with an RsiG homolog using the homolog from each individual genome as query and an e-value cutoff of 1 e-3. If multiple hits occurred in a genome, each hit was used as a query in a BLAST search of the National Center for Biotechnology Information (NCBI) database, as well as of the *S. venezuelae* genome (http://strepdb.streptomyces.org.uk/). Only one of these reciprocal searches resulted in hits to RsiG sequences, that for Acel_1994 of *A. cellulolyticus* 11B. In order to generate sequence logos, all 135 RsiG homologs were aligned using MUSCLE (49). Regions of the alignment homologous to RsiG_Sv_ α1 and α5 were extracted, gaps removed, and the resulting alignments submitted to https://weblogo.berkeley.edu/logo.cgi (50).

### Actinobacterial Phylogeny.

Amino acid sequences of 37 conserved housekeeping genes were automatically identified, aligned, and concatenated using Phylosift (51). Model selection was performed using SMS (52) implemented at http://www.atgc-montpellier.fr/phyml/ (53), which resulted in selection of a LG substitution model with γ-distributed rate variation between sites. Phylogenetic reconstruction was performed by RAxML version 8.2.10 (54) with 100 rapid bootstraps replicates to assess node support. The tree was visualized and formatted using iTOL (55). Taxonomic assignments were based on the taxonomy database maintained by the NCBI (https://www.ncbi.nlm.nih.gov/Taxonomy/Browser/wwwtax.cgi).

### Growth of *R. radiotolerans*, RNA Isolation, and 5′ Triphosphate End-Capture Sequencing.

*R. radiotolerans* was grown at 42 °C in Thermus 162 medium (https://www.dsmz.de/microorganisms/medium/pdf/DSMZ_Medium878.pdf) containing Trace Element Solution and 1% NaCl as both shaking and standing cultures. For each replicate, a shaking and standing culture were combined and cell pellets were washed with RNAprotect Bacteria Reagent (Qiagen). Pellets were resuspended in 900 µL lysis solution (400 µL phenol [pH4.3], 100 µL chlorophorm:isoamyl alcohol [24:1], and 400 µL RLT buffer [Qiagen]) with lysing matrix B (MP Biomedicals) and homogenized using an Omni Bead Ruptor 24 (Omni International). Lysates were cleared by centrifugation; the supernatants were removed and RNA was extracted using an RNEasy Kit (Qiagen) with on-column DNase I (Qiagen) digestion. A second DNase I treatment was carried out after extraction using a Turbo DNA-free Kit (Invitrogen). The 5′ triphosphate end-capture sequencing (Cappable-seq) was carried out by Vertis Biotechnologie, and genome-wide transcription start sites were identified by mapping the sequence reads onto the *R. radiotolerans* RSPS-4 reference genome sequence.

### Heterologous Complementation.

The *rsiG* gene from *R. radiotolerans* was amplified from genomic DNA using the RsiGRr forward and reverse primers and cloned under the control of the ermE* promoter in pIJ10257 (56), using the restriction enzymes NdeI and HindIII. The resulting plasmid, pIJ10947, was introduced into the *S. venezuelae rsiG* null mutant by conjugation from *E. coli*.

## Supplementary Material

Supplementary File

## Data Availability

Atomic coordinates and structure factor amplitudes for apo RsiG_Rr_ (crystal form 1), apo RsiG_Rr_ (crystal form 2), and *Rr* (RsiG)_2_–(c-di-GMP)_2_–WhiG have been deposited in the Protein Data Bank https://www.rcsb.org (PDB ID codes 7LQ2–7LQ4). All other study data are included in the article and *SI Appendix*.
