# Supplementary material for: Evolution of a σ–(c-di-GMP)–anti-σ switch
Source: Proc Natl Acad Sci U S A. 2021 Jul 21;118(30):e2105447118. doi: 10.1073/pnas.2105447118 (PMC8325347; doi:10.1073/pnas.2105447118)
Supplement: Supplementary File [file pnas.2105447118.sapp.pdf]

Supplementary Information for:

Evolution of a sigma-(c-di-GMP)-antisigma switch

Maria A. Schumacher[1,4], Kelley A. Gallagher[2,4,5], Neil A. Holmes[2,4], Govind Chandra[2], Max Henderson[1], David T. Kysela[3], Richard G. Brennan[1] and Mark J. Buttner[2]*

[1]Department of Biochemistry, Duke University School of Medicine, Durham, NC, USA

[2]Department of Molecular Microbiology, John Innes Centre, Norwich Research Park, Norwich NR4 7UH, UK

[3]Département de Microbiologie, Infectiologie et Immunologie, Université de Montréal, Montréal H3C 3J7, Canada

*Email: maria.schumacher@duke.edu or mark.buttner@jic.ac.uk

This PDF file includes:

Figures S1 to S9

Tables S1 to S3

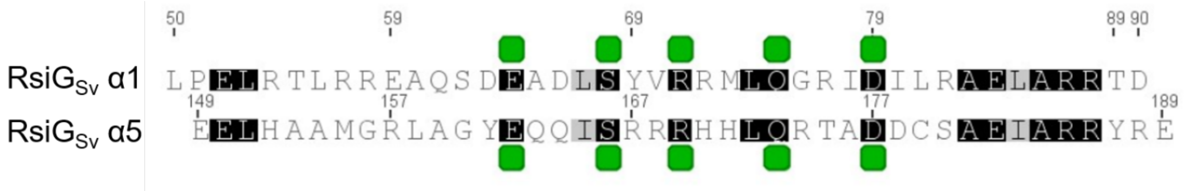

Fig. S1. Alignment of α1 and α5 of RsiG$_{Sv}$. Sequences were aligned and visualized using Geneious (http://www.geneious.com/). Green indicates the location of the c-di-GMP-binding residues.

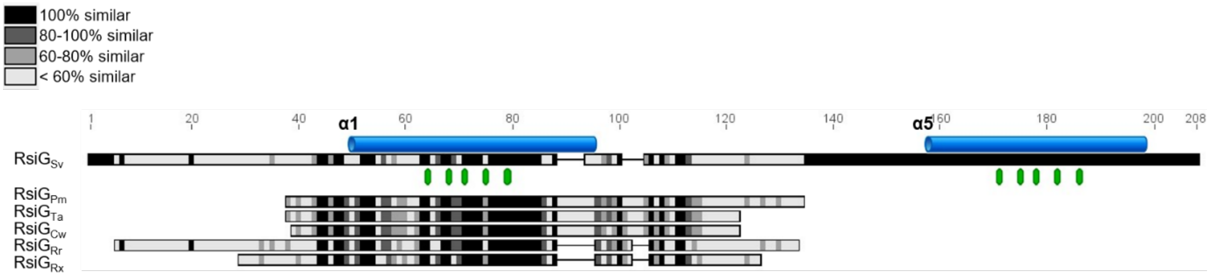

Fig. S2. Alignment of full length RsiG$_{Sv}$ with the five homologs where α5 is absent. Sequences were aligned and visualized using Geneious (http://www.geneious.com/). Blue indicates the location of the two c-di-GMP-binding α-helices in the RsiG$_{Sv}$, and green indicates the location of the c-di-GMP-binding residues. Residues are numbered based on the RsiG$_{Sv}$ sequence and colored based on similarity.

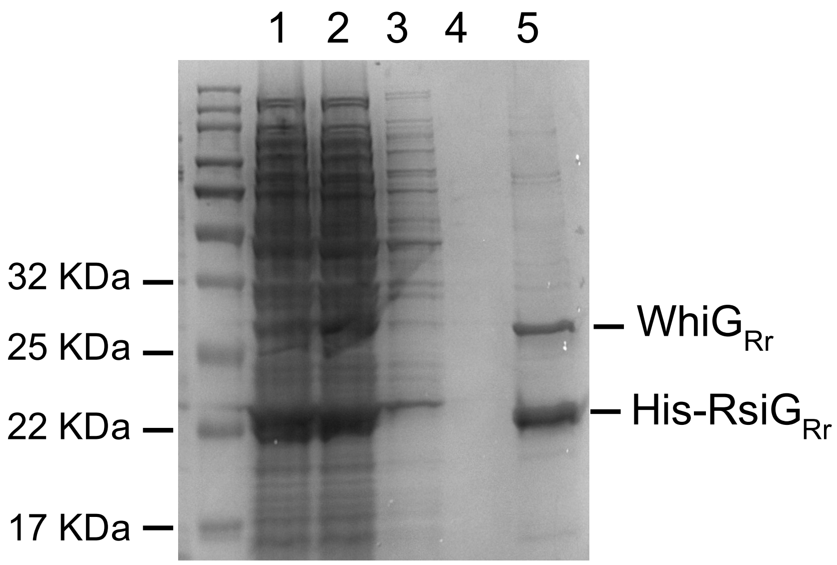

Fig. S3. The single-motif RsiG proteins form direct complexes with their cognate WhiG proteins. RsiG$_{Rr}$ + WhiG$_{Rr}$ were co-overexpressed in *E. coli* using pCOLADuet-1, with only RsiG$_{Rr}$ carrying a his-tag. Following co-overexpression, the soluble extract was passed over a nickel column and, after washing, bound proteins were eluted and analyzed on a 12% polyacrylamide-SDS gel.

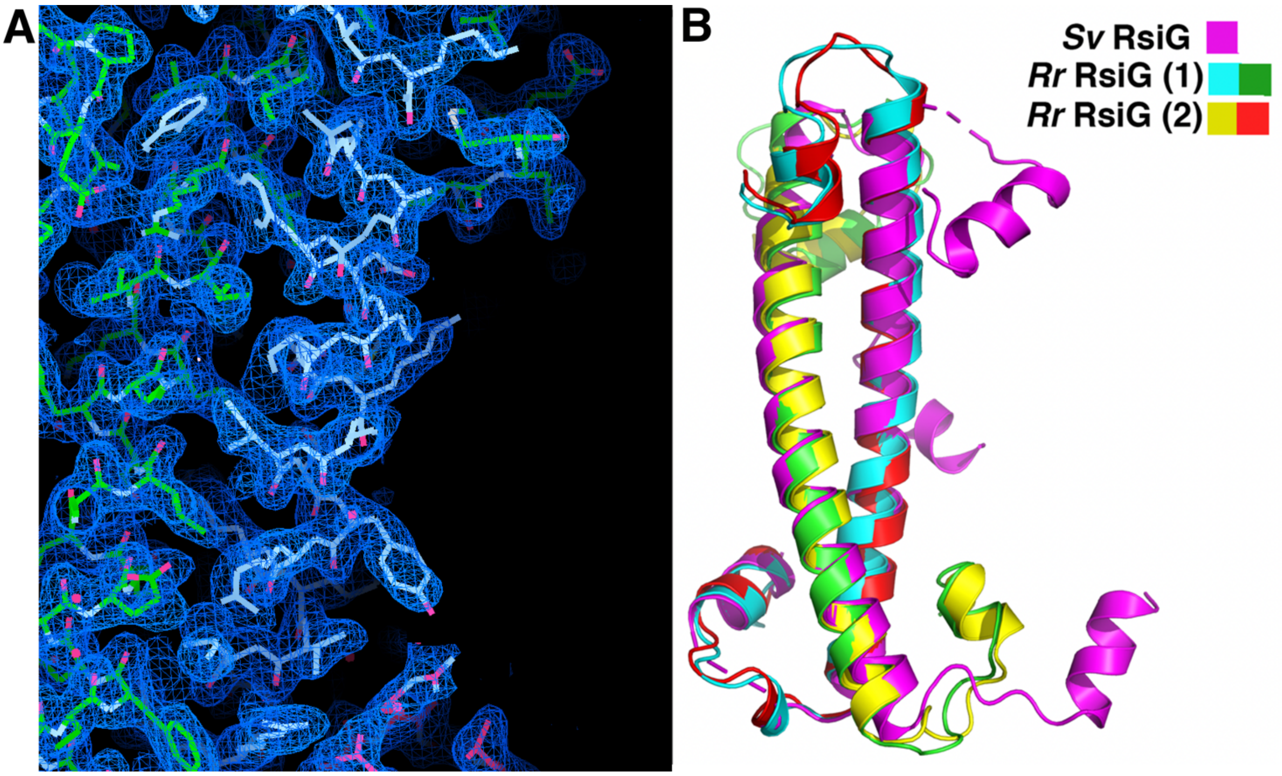

Fig. S4. **Apo RsiG$_{Rr}$ structure and comparison to the *Sv* RsiG structure from the *Sv* RsiG-(c-di-GMP)$_2$-WhiG complex**. (*A*) Section of the apo RsiG$_{Rr}$ (crystal form 1) experimental SAD electron density map (blue mesh), calculated to 1.86 Å and contoured at 1 σ. The final refined structure is shown as sticks. (*B*) Superimposition of apo *Rr* RsiG homodimers from crystal form 1 and crystal form 2 onto the structure of the *Sv* RsiG from the *Sv* RsiG-(c-di-GMP)$_2$-WhiG complex.

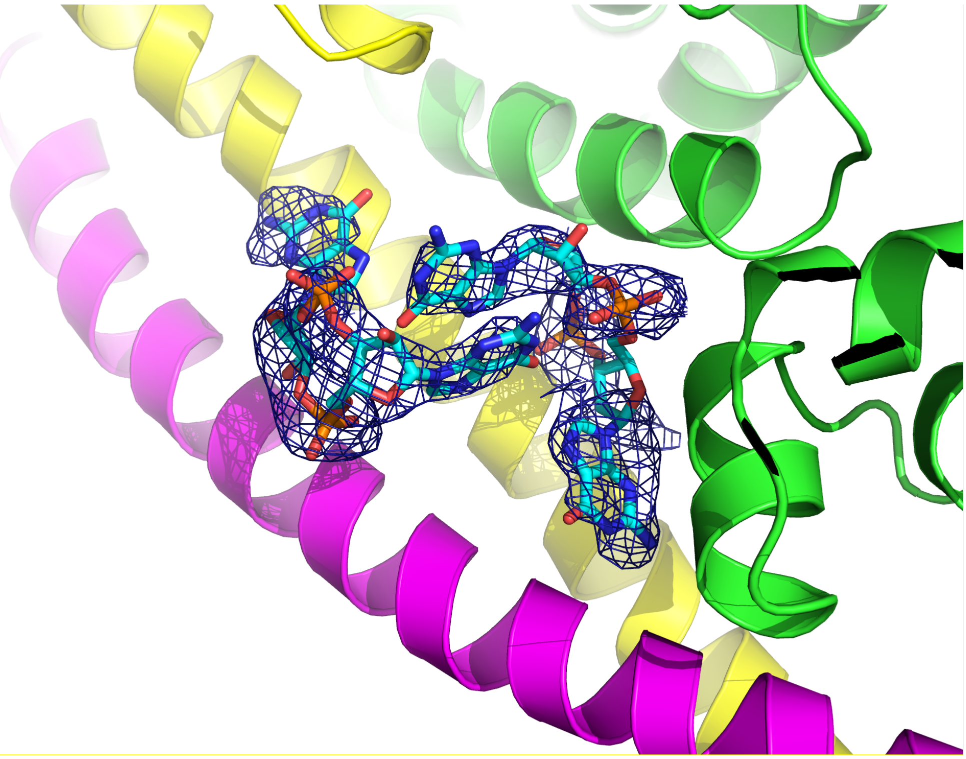

Fig. S5. Omit electron density for the c-di-GMP molecules in the *Rr* (RsiG)$_2$-(c-di-GMP)$_2$-WhiG structure. WhiG is shown in green and the two identical RsiG subunits are shown in magenta and yellow. The bound c-di-GMP dimer is shown as sticks, and an mFo-DFc map omit map, calculated in Phenix.refine, and contoured at 2.6 σ is shown as a blue mesh.

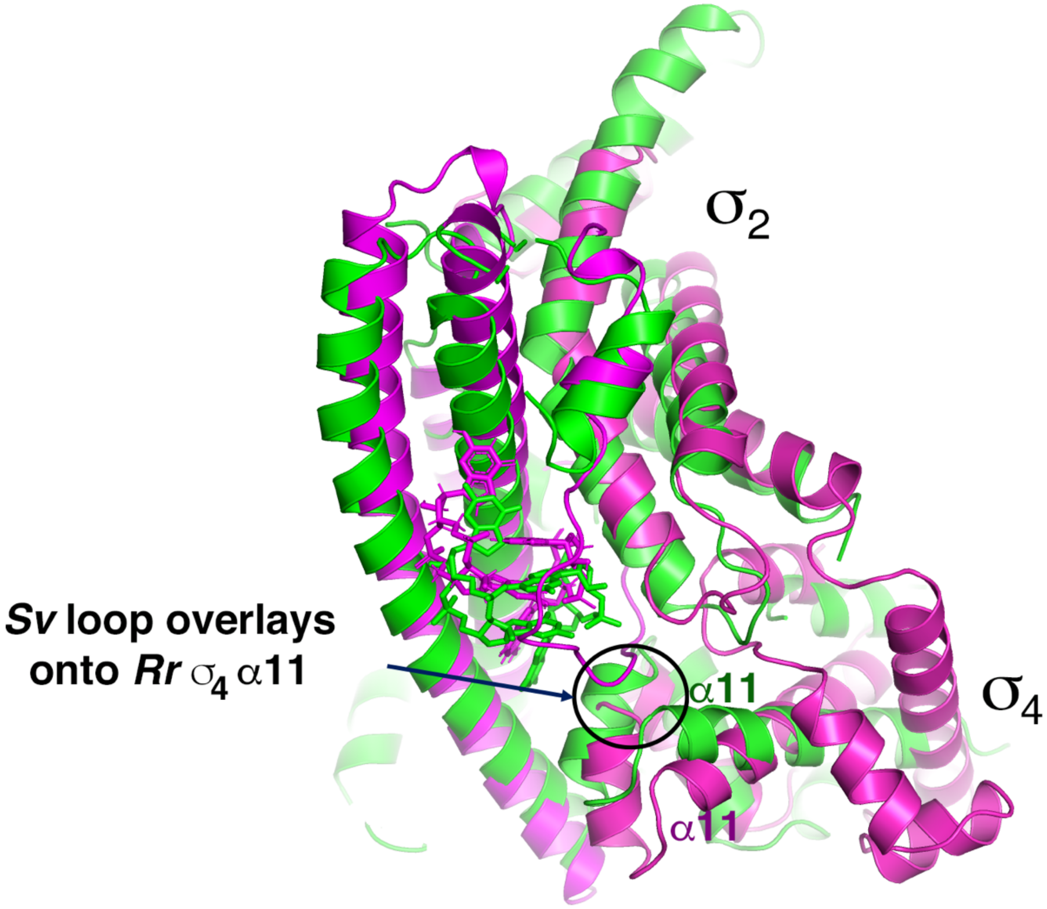

Fig. S6. Superimposition of the *Rr* (RsiG)$_2$-(c-di-GMP)$_2$-WhiG structure (green) onto the *Sv* RsiG-(c-di-GMP)$_2$-WhiG structure (magenta). The complexes are similar overall but notably shifted, however, are the σ_4 domains, where the position of *Rr* σ_4 is rotated by approximately 25º compared to *Sv* σ_4. As a result, the c-di-GMP-binding loop of *Sv* RsiG overlays the position of $WhiG_{Rr}$ σ_4.

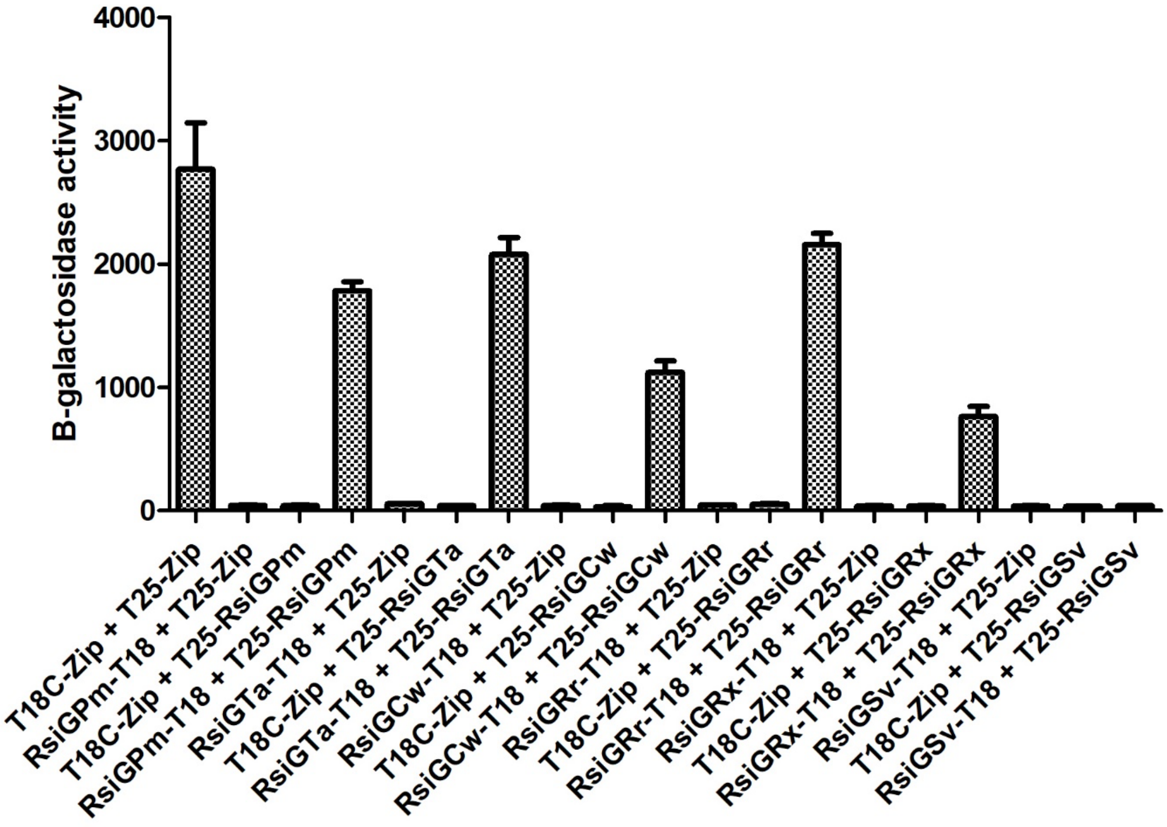

Fig. S7. Single-motif RsiG proteins self-interact in a BACTH assay, but RsiG$_{Sv}$ does not. Pm *Patulibacter medicamentivorans*; Ta *Thermoleophilum album*; Cw *Conexibacter woesei*; Rr *Rubrobacter radiotolerans*; Rx *Rubrobacter xylanophilus*; Sv *Streptomyces venezuelae*. The listed pairs of constructs were transferred into the BACTH reporter strain *E. coli* BTH101 by transformation. The resulting transformants were selected on LB agar containing 100 µg/ml carbenicillin and 50 µg/ml kanamycin and incubated at 37°C before single colonies were picked and subjected to β-galactosidase assays. Strains expressing fusions of both adenylate cyclase domains to the leucine zipper domain of GCN4 (zip) served as a positive control, while strains expressing fusions of one adenylate cyclase domain to a zip domain and the other to RsiG served as negative controls. Results are the average of three replicate cultures derived from the same single colony. Error bars represent the SEM.

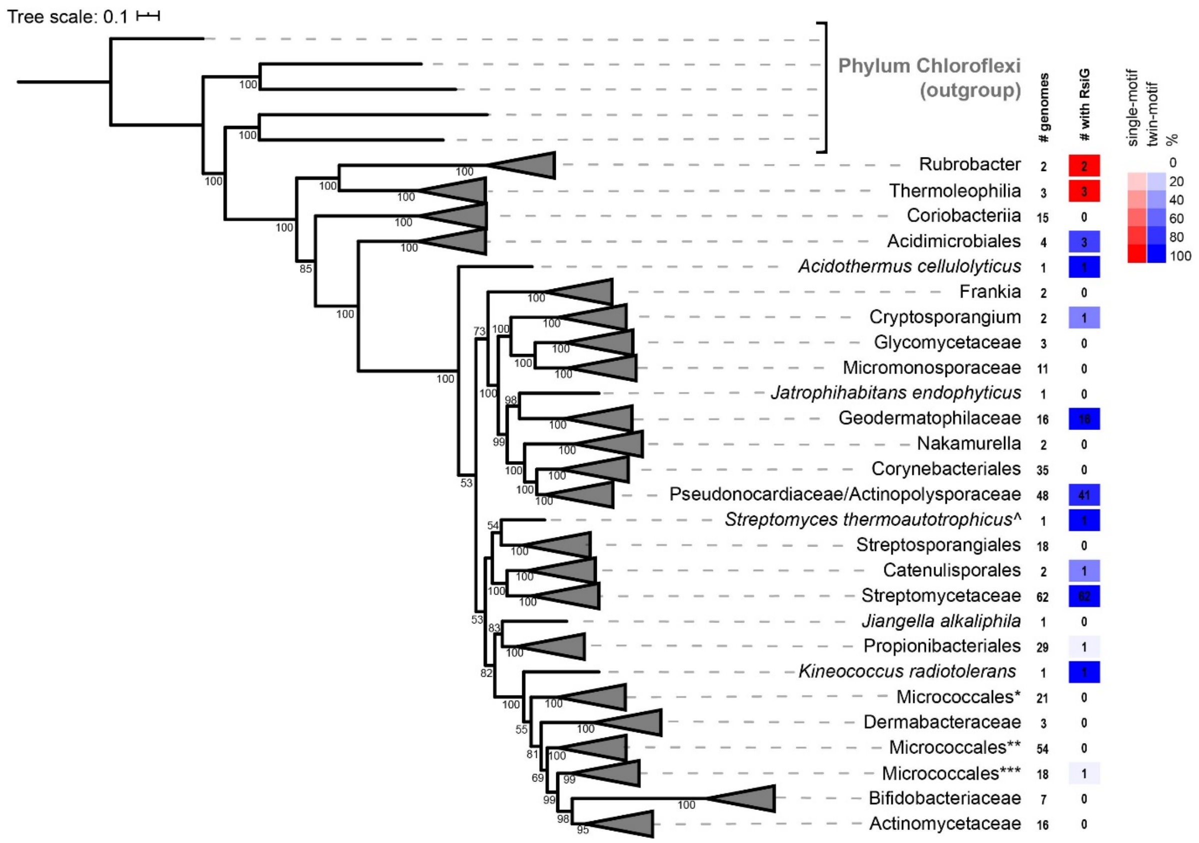

Fig. S8. Distribution of RsiG in the Phylum Actinobacteria. Maximum likelihood phylogeny based on a concatenated alignment of 37 housekeeping genes from 378 representative Actinobacterial genomes. Five genomes from Phylum Chloroflexi are included as outgroups. Bootstrap values ≥50 are indicated at their respective nodes (based on 100 replicates). Triangles are used to represent where clades have been collapsed. For each collapsed clade, the total number of genomes and the number of genomes possessing an RsiG homolog are provided. Heatmaps represent the percentage of total genomes within each clade possessing a single-motif (red) or twin-motif (blue) RsiG homolog. Tree scale is substitutions per site. "*Streptomyces thermoautotrophicus*" (indicated by a ^) was originally classified as a member of the genus *Streptomyces*, but after genome sequencing it was found to be a non-streptomycete most closely related to Streptosporangiales (1). It has yet to be renamed. *Includes Micrococcales families Dermatophilaceae, Intrasporangiaceae, Dermacoccaceae. **Includes Micrococcales families Brevibacteriaeae, Micrococcaceae, and Microbacteriaeae. ***Includes Micrococcales families Promicromonosporaceae, Cellulomonadaceae, Sanguibacteraceae, Jonesiaceae, Ruaniaceae, and Beutenbergiaceae.

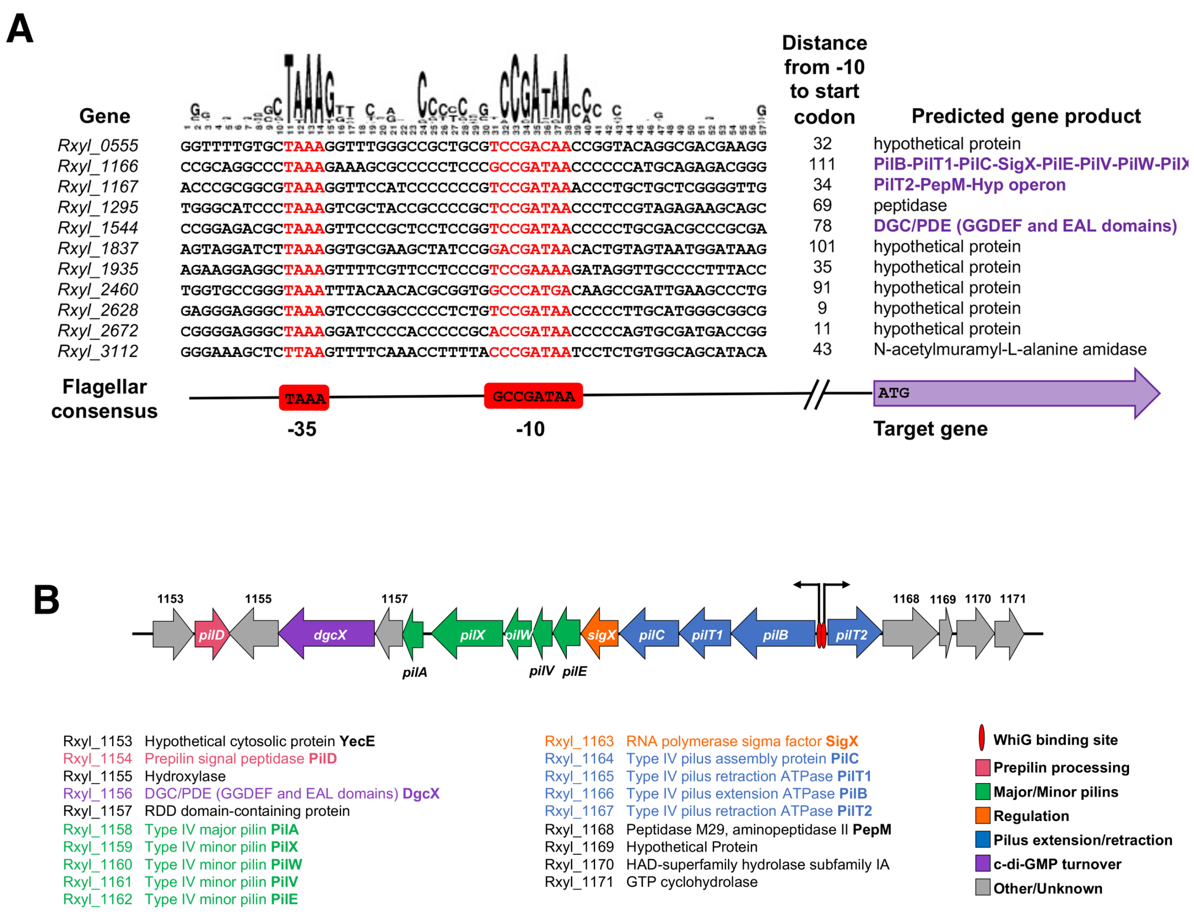

Fig. S9. Predicted WhiG target promoters in *R. xylanophilus* and organization of the *R. xylanophilus* type IV pilus gene cluster, showing the positions of predicted WhiG target promoters. (*A*) Eleven matches to the well-established ‘flagellar’ promoter consensus sequence (-35 TAAA; -10 GCCGATAA; [2]) were identified bioinformatically in the intergenic regions of the *R. xylanophilus* genome, lying within 200 bp of a downstream start codon, and allowing for up to two base mismatches in total. Putative -10 and -35 sequences are shown in red. Target genes with predicted functions in type IV pilus biosynthesis or c-di-GMP turnover are highlighted in purple. The Logo based on the sequence alignment was created using Weblogo (3). (*B*) The genes in the type IV pilus gene cluster are shown as schematics with the predicted gene products listed below with the same color coding. Note the presence of *dgcX*, a gene encoding a DGC/PDE enzyme, embedded within the type IV pilus gene cluster.

Table S1: RsiG homologs identified in this study

Organism	NCBI Taxonomic ID	Family	RsiG Gene ID
Acidithrix ferrooxidans	1280514	Acidimicrobiaceae	AXFE_25810
Acidothermus cellulolyticus 11B	351607	Acidothermaceae	Acel_0065
Acidothermus cellulolyticus 11B	351607	Acidothermaceae	Acel_1994
Actinoalloteichus hoggarensis	1470176	Pseudonocardiaceae	AHOG_26940
Actinoalloteichus hymeniacidonis	340345	Pseudonocardiaceae	TL08_25965
Actinokineospora bangkokensis	1193682	Pseudonocardiaceae	BJP25_30305
Actinokineospora spheciospongiae	909613	Pseudonocardiaceae	UO65_2108
Actinokineospora terrae	155974	Pseudonocardiaceae	SAMN04487818_109182
Actinophytocola xanthii	1912961	Pseudonocardiaceae	BU204_35140
Actinophytocola xinjiangensis	485602	Pseudonocardiaceae	BLA60_20395
Actinopolyspora righensis	995060	Actinopolysporaceae	SAMN04487904_104350
Actinopolyspora xinjiangensis	405564	Actinopolysporaceae	SAMN04487905_109114
Actinosynnema mirum DSM 43827	446462	Pseudonocardiaceae	Amir_6839
Alloactinosynnema album	504798	Pseudonocardiaceae	SAMN05421871_106253
Alloactinosynnema iranicum	1271860	Pseudonocardiaceae	SAMN05216174_105197
Allokutzneria albata	211114	Pseudonocardiaceae	SAMN04489726_5420
Amycolatopsis australiensis	546364	Pseudonocardiaceae	SAMN04489730_1567
Amycolatopsis japonica	208439	Pseudonocardiaceae	AJAP_40520
Amycolatopsis marina	490629	Pseudonocardiaceae	SAMN05216266_101214
Amycolatopsis mediterranei U32	749927	Pseudonocardiaceae	AMED_9021
Amycolatopsis methanolica 239	1068978	Pseudonocardiaceae	AMETH_6908
Amycolatopsis rubida	112413	Pseudonocardiaceae	SAMN05421854_1011294
Amycolatopsis saalfeldensis	394193	Pseudonocardiaceae	SAMN04489732_119183
Amycolatopsis sacchari	115433	Pseudonocardiaceae	SAMN05421835_10624
Amycolatopsis xylanica	589385	Pseudonocardiaceae	SAMN05421504_102620
Blastococcus endophyticus	673521	Geodermatophilaceae	SAMN05660991_01077
Blastococcus saxobsidens DD2	1146883	Geodermatophilaceae	BLASA_4514
Catenulispora acidiphila DSM 44928	479433	Catenulisporaceae	Caci_8239
Cellulomonas gilvus ATCC 13127	593907	Cellulomonadaceae	Celgi_2837
Conexibacter woesei DSM 14684	469383	Conexibacteraceae	Cwoe_3492
Cryptosporangium aurantiacum	134849	Cryptosporangiaceae	SAMN05443668_112227
Ferrithrix thermotolerans DSM 19514	1121881	Acidimicrobiaceae	SAMN02745225_00312
Geodermatophilus africanus	1137993	Geodermatophilaceae	SAMN05660209_02770
Geodermatophilus amargosae	1296565	Geodermatophilaceae	SAMN05660657_01019
Geodermatophilus nigrescens	1070870	Geodermatophilaceae	SAMN05444351_3171
Geodermatophilus obscurus	1861	Geodermatophilaceae	SAMN05660359_01819
Geodermatophilus obscurus DSM 43160	526225	Geodermatophilaceae	Gobs_4624
Geodermatophilus pulveris	1564159	Geodermatophilaceae	SAMN06893096_10164
Geodermatophilus ruber	504800	Geodermatophilaceae	SAMN04488085_101205
Geodermatophilus saharensis	1137994	Geodermatophilaceae	SAMN04488107_0150
Geodermatophilus siccatus	1137991	Geodermatophilaceae	SAMN05660642_04689
Geodermatophilus telluris	1190417	Geodermatophilaceae	SAMN05660690_0674
Haloechinothrix alba	664784	Pseudonocardiaceae	SAMN06265360_105203
Ilumatobacter coccineus YM16-304	1313172	Ilumatobacteraceae	YM304_37910
Kibdelosporangium aridum	2030	Pseudonocardiaceae	SAMN05661093_02960
Kibdelosporangium phytohabitans	860235	Pseudonocardiaceae	AOZ06_51490
Kineococcus radiotolerans SRS30216	266940	Kineosporiaceae	Krad_0885
Kitasatospora aureofaciens	1894	Streptomycetaceae	B6264_09810
Kitasatospora cheerisanensis KCTC 2395	1348663	Streptomycetaceae	KCH_43270

Kitasatospora setae KM-6054	452652	Streptomycetaceae	KSE_43740
Klenkia soli	1052260	Geodermatophilaceae	SAMN05660199_04463
Klenkia taihuensis	1225127	Geodermatophilaceae	SAMN05661030_0947
Kutzneria albida DSM 43870	1449976	Pseudonocardiaceae	KALB_8526
Lentzea albida	65499	Pseudonocardiaceae	SAMN04488000_108266
Lentzea guizhouensis	1586287	Pseudonocardiaceae	BBK82_16140
Modestobacter caceresii	1522368	Geodermatophilaceae	IN07_14980
Modestobacter marinus	477641	Geodermatophilaceae	MODMU_5029
Nocardioides psychrotolerans	1005945	Nocardioidaceae	SAMN05216561_101412
Patulibacter medicamentivorans	1097667	Patulibacteraceae	PAI11_41790
Prauserella marina	530584	Pseudonocardiaceae	BAY61_30440
Rubrobacter radiotolerans	42256	Rubrobacteraceae	RradSPS_1442
Rubrobacter xylanophilus DSM 9941	266117	Rubrobacteraceae	Rxyl_1435
Saccharomonospora azurea NA-128	882081	Pseudonocardiaceae	SacazDRAFT_02570
Saccharomonospora cyanea NA-134	882082	Pseudonocardiaceae	SaccyDRAFT_4973
Saccharomonospora glauca K62	928724	Pseudonocardiaceae	SacglDRAFT_04182
Saccharomonospora marina XMU15	882083	Pseudonocardiaceae	SacmaDRAFT_5517
Saccharomonospora viridis DSM 43017	471857	Pseudonocardiaceae	Svir_37760
Saccharomonospora xinjiangensis XJ-54	882086	Pseudonocardiaceae	SacxiDRAFT_2281
Saccharopolyspora erythraea NRRL 2338	405948	Pseudonocardiaceae	SACE_7115
Saccharopolyspora flava	95161	Pseudonocardiaceae	SAMN05660874_03916
Saccharopolyspora jiangxiensis	418522	Pseudonocardiaceae	SAMN05216506_12021
Saccharothrix espanaensis DSM 44229	1179773	Pseudonocardiaceae	BN6_82120
Streptoalloteichus hindustanus	2017	Pseudonocardiaceae	SAMN05444320_101335
Streptomyces aidingensis	910347	Streptomycetaceae	SAMN05421773_102489
Streptomyces albireticuli	1940	Streptomycetaceae	SMD11_2948
Streptomyces alboflavus	67267	Streptomycetaceae	SMD44_03945
Streptomyces albus	1888	Streptomycetaceae	SLNWT_4079
Streptomyces alfalfa	1642299	Streptomycetaceae	A7J05_19230
Streptomyces alni	380248	Streptomycetaceae	SAMN05216251_12497
Streptomyces auratus AGR0001	1160718	Streptomycetaceae	SU9_11895
Streptomyces autolyticus	75293	Streptomycetaceae	BV401_24975
Streptomyces avermitilis MA-4680	227882	Streptomycetaceae	SAVERM_4026
Streptomyces azureus	146537	Streptomycetaceae	SAZU_3947
Streptomyces bingchenggensis BCW-1	749414	Streptomycetaceae	SBI_05067
Streptomyces caatingaensis	1678637	Streptomycetaceae	AC230_13490
Streptomyces canus	58343	Streptomycetaceae	AQI96_41900
Streptomyces cattleya NRRL 8057	1003195	Streptomycetaceae	SCATT_25300
Streptomyces clavuligerus	1901	Streptomycetaceae	BB341_12590
Streptomyces coelicolor A3(2)	100226	Streptomycetaceae	SCO4184
Streptomyces collinus Tu 365	1214242	Streptomycetaceae	B446_17940
Streptomyces davaonensis JCM 4913	1214101	Streptomycetaceae	BN159_4809
Streptomyces gilvigriseus	1428644	Streptomycetaceae	BIV57_09550
Streptomyces glaucescens	1907	Streptomycetaceae	SGLAU_15805
Streptomyces glauciniger	235986	Streptomycetaceae	SAMN05216252_124117
Streptomyces griseoaurantiacus M045	996637	Streptomycetaceae	SGM_1517
Streptomyces griseochromogenes	68214	Streptomycetaceae	AVL59_45420
Streptomyces griseus subsp. griseus NBRC 13350	455632	Streptomycetaceae	SGR_3974
Streptomyces guanduensis	310781	Streptomycetaceae	SAMN05216259_12052
Streptomyces himastatinicus ATCC 53653	457427	Streptomycetaceae	SSOG_04022
Streptomyces indicus	417292	Streptomycetaceae	SAMN05421806_11878
Streptomyces jeddahensis	1716141	Streptomycetaceae	STSP_33290
Streptomyces leeuwenhoekii	1437453	Streptomycetaceae	sle_38330
Streptomyces lincolnensis	1915	Streptomycetaceae	SLINC_3946

Streptomyces lydicus	47763	Streptomycetaceae	SL103_32765
Streptomyces melanosporofaciens	67327	Streptomycetaceae	SAMN04490356_3817
Streptomyces mobaraensis NBRC 13819	1223523	Streptomycetaceae	H340_21216
Streptomyces nanshensis	518642	Streptomycetaceae	AN218_23890
Streptomyces niveus	193462	Streptomycetaceae	BBN63_18595
Streptomyces nodosus	40318	Streptomycetaceae	SNOD_15860
Streptomyces noursei ATCC 11455	316284	Streptomycetaceae	SNOUR_18455
Streptomyces oceani	1075402	Streptomycetaceae	AN216_26240
Streptomyces olivochromogenes	1963	Streptomycetaceae	AQJ27_22315
Streptomyces paucisporeus	310782	Streptomycetaceae	SAMN05216499_12256
Streptomyces pluripotens	1355015	Streptomycetaceae	LK06_017615
Streptomyces pristinaespiralis	38300	Streptomycetaceae	SPRI_4030
Streptomyces puniciscabiei	164348	Streptomycetaceae	BFF78_22920
Streptomyces qinglanensis	943816	Streptomycetaceae	SAMN05421870_11845
Streptomyces radiopugnans	403935	Streptomycetaceae	SAMN05216481_10549
Streptomyces rubidus	310780	Streptomycetaceae	SAMN05216267_102496
Streptomyces scabiei 87.22	680198	Streptomycetaceae	SCAB_49711
Streptomyces thermoautotrophicus	1469144	unassigned	TH66_22085
Streptomyces thermolilacinus SPC6	1306406	Streptomycetaceae	J116_012715
Streptomyces tsukubensis	83656	Streptomycetaceae	B1H18_15915
Streptomyces tsukubensis NRRL18488	1114943	Streptomycetaceae	STSU_19180
Streptomyces turgidiscabies Car8	698760	Streptomycetaceae	STRTUCAR8_06016
Streptomyces uncialis	1048205	Streptomycetaceae	AB852_36725
Streptomyces venezuelae	54571	Streptomycetaceae	AQF52_4351 (vnz19430)
Streptomyces vietnamensis	362257	Streptomycetaceae	SVTN_20555
Streptomyces viridochromogenes DSM 40736	591159	Streptomycetaceae	SSQG_03626
Streptomyces viridosporus ATCC 14672	566461	Streptomycetaceae	SSFG_03876
Streptomyces wuyuanensis	1196353	Streptomycetaceae	SAMN05444921_12880
Streptomyces xiamenensis	408015	Streptomycetaceae	SXIM_30820
Streptomyces zinciresistens K42	700597	Streptomycetaceae	SZN_12578
Thermoleophilum album	29539	Thermoleophilaceae	SAMN02745716_1666
Yuhushiella deserti	587909	Pseudonocardiaceae	SAMN05421810_103293

Table S2: Data collection and refinement statistics: *Rr* RsiG and *Rr* $(RsiG)_2$-(c-di-GMP)$_2$-WhiG complex

	Rr RsiG crystal form 1	*Rr* RsiG crystal form 2	*Rr* $(RsiG)_2$-(c-di-GMP)$_2$-WhiG
**Pdb code**	7LQ2	7LQ3	7LQ4
**Data collection**			
Space group	$P22_12_1$	$P4_32_12$	$P2_12_12_1$
Cell dimensions			
a, *b*, *c* (Å)	59.48, 95.29,117.60	78.54, 78.54,81.34	46.47,81.62,114.59
α, β, γ (°)	90.0,90.0,90.0	90.0,90.0,90.0	90.0,90.0,90.0
Resolution (Å)	50.46 - 1.86 (1.92-1.86)*	39.27 - 2.55 (2.59-2.55)	66.48 - 2.93 (3.04-2.93)
R_{sym}	0.053 (0.464)	0.087 (1.056)	0.043 (0.240)
R_{pim}	0.032 (0.374)	0.028 (0.453)	0.035 (0.235)
$I/\sigma I$	17.8 (2.4)	20.2 (2.9)	17.1 (4.4)
Completeness (%)	99.6 (99.6)	99.6 (100.0)	91.9 (63.7)
Redundancy	6.6 (5.9)	10.5 (10.0)	2.9 (2.6)
CC(1/2)	0.998 (0.898)	0.999 (0.889)	0.999 (0.804)
**Refinement**			
Resolution (Å)	50.46 - 1.86	39.27 - 2.55	66.48 - 2.93
No. reflections	57371 (5313)	8738 (847)	9237 (601)
R_{work} / R_{free}	17.3%/20.2%	19.9%/26.8%	26.5%/27.9%
No. atoms			
Protein	3473	1701	2667
Ligand/ion	10	12	92
Water	457	213	12
B-factors			
Protein	25.7	64.3	94.9
Ligand/ion	15.7	64.2	72.1
Water	39.5	88.7	75.2
R.m.s. deviations			
Bond lengths (Å)	0.015	0.006	0.003
Bond angles (°)	1.30	0.86	0.910
Ramachandran analyses			
Favored (%)	98.6	98.1	95.8
Disallowed (%)	0.0	0.0	0.0
MolProbity score	1.34	2.04	2.44

*Values in parentheses are for highest-resolution shell.

Table S3: Primers, plasmids, and strains used in this study

Primers	5′ Sequence
BACTH_rsiGTa_Fwd	GGATCATCTAGAGATGATGGACACCTTCCCGGA
BACTH_rsiGTa_Rev	CGATCAGGTACCCGGCCTTCATCGTCCGGCAGGC
BACTH_rsiGRr_Fwd	GGATCATCTAGAGATGGCGAGGAAACCTACGAGGG
BACTH_rsiGRr_Rev	CGATCAGGTACCCGCGCGCCATCACCACGACGGT
BACTH_rsiGCw_Fwd	GGATCATCTAGAGATGGACACCTTCCCGGATCT
BACTH_rsiGCw_Rev	CGATCAGGTACCCGGTTCGGCTCGCCACCCGGCAC
BACTH_rsiGPm_Fwd	GGATCATCTAGAGATGGAAGACACCTTCCCGGATC
BACTH_rsiGPm_Rev	CGATCAGGTACCCGGCTGGTCGCGCTGCCCTCGC
BACTH_rsiGRx_Fwd	GGATCATCTAGAGATGGCGAGCTGGAACGTGGTG
BACTH_rsiGRx_Rev	CGATCAGGTACCCGACGGTGACGACGGCCACCTTC
RsiGTa_MCS1_thromb_Fwd	GAGTCAGAATTCGCTGGTGCCGCGCGGCAGCATGATGGACACCTTCCCGGATC
RsiGTa_MCS1_Rev	GAGTCAAAGCTTTTAGCCTTCATCGTCCGGCAGGC
WhiGTa_MCS2_Fwd	GAGTCACATATGGAGACCCACCTGAAGGAG
WhiGTa_MCS2_Rev	GAGTCAGGTACCTTATTCACCATCGTCGCCCTCGC
RsiGRr_MCS1_thromb_Fwd	GAGTCAGAATTCGCTGGTGCCGCGCGGCAGCATGGGCGAGGAAACCTACGAG
RsiGRr_MCS1_Rev	GAGTCAAAGCTTTTACGCGCCATCACCACGACGGTC
WhiGRr_MCS2_Fwd	GAGTCACATATGCGTGTTAGCATCGAACGTCTG
WhiGRr_MCS2_Rev	GAGTCAGGTACCTTAGCTGGTACGCGGCTCGCTCAG
RsiGCw_MCS1_thromb_Fwd	GAGTCAGAATTCGCTGGTGCCGCGCGGCAGCATGGACACCTTCCCGGATCTGG
RsiGCw_MCS1_Rev	GAGTCAAAGCTTTTAGTTCGGCTCGCCACCCGGCAC
WhiGCw_MCS2_Fwd	GAGTCACATATGGAGACCAACGTGAAGGC
WhiGCw_MCS2_Rev	GAGTCAGGTACCTTAGCCGTTATCCGCGTCTTCGT
RsiGPm_MCS1_thromb_Fwd	GAGTCAGAATTCGCTGGTGCCGCGCGGCAGCATGGAAGACACCTTCCCGGATC
RsiGPm_MCS1_Rev	GAGTCAAAGCTTTTAGCTGGTCGCGCTGCCCTCGC
WhiGPm_MCS2_Fwd	GAGTCACATATGGCGATCACCCACGAGACCCGTG
WhiGPm_MCS2_Rev	GAGTCAGGTACCTTACGCGTTTTCAACATCGCCAC
RsiGRx_MCS1_thromb_Fwd	GAGTCAGAATTCGCTGGTGCCGCGCGGCAGCATGGGCGAGCTGGAACGTGGTG
RsiGRx_MCS1_Rev	GAGTCAAAGCTTTTAACGGTGACGACGGCCACCTTC
WhiGRx_MCS2_Fwd	GAGTCACATATGAGCGGTCCGACCCTGGGCCGTC
WhiGRx_MCS2_Rev	GAGTCAGGTACCTTAAACACCGCTCAGACGATCAC
RsiGRr-nde-Fwd	CTAGCACATATGGGTGAAGAGACTTATG
RsiGRr-hind-Rev	CAGCCTAAGCTTTCAAGCTCCATCACCCCGGCGATC

Plasmid	Relevant Genotype/comments	Source or reference
pUT18	Two-hybrid plasmid, C-terminal cyaAT18fusion (AmpR)	4
pKT25	Two-hybrid plasmid, N-terminal cyaAT25 fusion (KanR)	4
pUT18C-zip	A derivative of pUT18C in which the leucine zipper of GCN4 is genetically fused in-frame to the T18 fragment	4
pKT25-zip	A derivative of pKT25 in which the leucine zipper of GCN4 is genetically fused in frame to the T25 fragment	4
pIJ10911	pKT25 carrying *rsiGSv*	5
pIJ10912	pUT18 carrying *rsiGSv*	5
pCOLADuet1	Expression vector for coexpression of two target genes, each under the control of a T7 promoter (KanR)	Novagen
pIJ10257	φBT1 attP-int derived integration vector for the conjugal transfer of DNA from *E. coli* to *Streptomyces* (HygR) with the *ermE** promoter driving constitutive expression	6
pIJ10927	pUT18 carrying *rsiGTa* (gene codon optimised for *E.coli*)	This work
pIJ10928	pKT25 carrying *rsiGTa* (gene codon optimised for *E.coli*)	This work
pIJ10929	pUT18 carrying *rsiGRr* (gene codon optimised for *E.coli*)	This work
pIJ10930	pKT25 carrying *rsiGRr* (gene codon optimised for *E.coli*)	This work
pIJ10931	pUT18 carrying *rsiGCw* (gene codon optimised for *E.coli*)	This work
pIJ10932	pKT25 carrying *rsiGCw* (gene codon optimised for *E.coli*)	This work
pIJ10933	pUT18 carrying *rsiGPm* (gene codon optimised for *E.coli*)	This work

pIJ10934	pKT25 carrying *rsiGPm* (gene codon optimised for *E.coli*)	This work
pIJ10935	pUT18 carrying *rsiGRx* (gene codon optimised for *E.coli*)	This work
pIJ10936	pKT25 carrying *rsiGRx* (gene codon optimised for *E.coli*)	This work
pIJ10937	pCOLADuet1 carrying *rsiGTa* at MCS1 (gene codon optimised for *E.coli*)	This work
pIJ10938	pCOLADuet1 carrying *rsiGTa* at MCS1 and *whiGTa* at MCS2 (gene codon optimised for *E.coli*)	This work
pIJ10939	pCOLADuet1 carrying *rsiGRr* at MCS1 (gene codon optimised for *E.coli*)	This work
pIJ10940	pCOLADuet1 carrying *rsiGRr* at MCS1 and *whiGRr* at MCS2 (gene codon optimised for *E.coli*)	This work
pIJ10941	pCOLADuet1 carrying *rsiGCw* at MCS1 (gene codon optimised for *E.coli*)	This work
pIJ10942	pCOLADuet1 carrying *rsiGCw* at MCS1 and *whiGCw* at MCS2 (gene codon optimised for *E.coli*)	This work
pIJ10943	pCOLADuet1 carrying *rsiGPm* at MCS1 (gene codon optimised for *E.coli*)	This work
pIJ10944	pCOLADuet1 carrying *rsiGPm* at MCS1 and *whiGPm* at MCS2 (gene codon optimised for *E.coli*)	This work
pIJ10945	pCOLADuet1 carrying *rsiGRx* at MCS1 (gene codon optimised for *E.coli*)	This work
pIJ10946	pCOLADuet1 carrying *rsiGRx* at MCS1 and *whiGPm* at MCS2 (gene codon optimised for *E.coli*)	This work
pIJ10947	pIJ10257 carrying *rsiGRr* under the control of the *ermE** promoter	This work

**Strains**	**Relevant Genotype/comments**	**Source or reference**
DH5α	F– φ80*lacZ*ΔM15 Δ(*lac*ZYA-*arg*F)U169 *rec*A1 *end*A1 *hsd*R17(rK–, mK+) *pho*A *sup*E44 λ– *thi*-1 *gyr*A96 *rel*A1	Invitrogen
BL21(DE3) pLysS	F– *ompT hsdSB*(rB– mB–) *gal dcm* (DE3), pLysS(cmR)	7
C41(DE3)	F– *ompT hsdSB*(rB– mB–) *gal dcm* (DE3)	8
BTH101	F– *cya-99 araD139 galE15 galK16 rpsL1 (Strr) hsdR2 mcrA1 mcrB1*	4
Rubrobacter radiotolerans RSPS-4	Wild type	Spanish Type Culture Collection of Microorganisms (CECT) No. 8386

SI References

1. D. MacKellar *et al*., *Streptomyces thermoautotrophicus* does not fix nitrogen. *Sci Rep*. **6**, 20086 (2016).
2. J.D. Helmann, Alternative sigma factors and the regulation of flagellar gene expression. *Mol. Microbiol.* **5**, 2875-82 (1991).
3. G.E. Crooks, G. Hon, J.M. Chandonia, S.E. Brenner, WebLogo: A sequence logo generator. Genome Res. **14**, 1188-1190 (2004).
4. G. Karimova, J. Pidoux, A. Ullmann, D. Ladant, A bacterial two-hybrid system based on a reconstituted signal transduction pathway. *Proc. Natl. Acad. Sci. USA* **95**, 5752–5756 (1998).
5. K.A Gallagher *et al*., c-di-GMP arms an anti-σ to control progression of multicellular differentiation in *Streptomyces*. *Mol. Cell* **77**, 586-599 (2020).
6. H.-J. Hong, M.I. Hutchings, L.M. Hill, M.J. Buttner, The role of the novel Fem protein VanK in vancomycin resistance in *Streptomyces coelicolor*. *J. Biol Chem.* **280,** 13055-13061 (2005).
7. F.W. Studier, B.A. Moffatt, Use of bacteriophage T7 RNA polymerase to direct selective high-level expression of cloned genes. *J. Mol. Biol.* **189,** 113-30 (1986).
8. S. Schlegel, P. Genevaux, J.W. de Gier, De-convoluting the genetic adaptations of *E. coli* C41(DE3) in real time reveals how alleviating protein production stress improves yields. *Cell Rep.* **10,** 1758-1766 (2015).
